# Genetic Evidence for Distinct Functions of Peptidoglycan Endopeptidases in *Escherichia coli*

**DOI:** 10.3389/fmicb.2020.565767

**Published:** 2020-09-11

**Authors:** Si Hyoung Park, Yung Jae Kim, Han Byeol Lee, Yeong-Jae Seok, Chang-Ro Lee

**Affiliations:** ^1^Department of Biological Sciences and Bioinformatics, Myongji University, Yongin, South Korea; ^2^Department of Biological Sciences and Institute of Microbiology, Seoul National University, Seoul, South Korea

**Keywords:** peptidoglycan, peptidoglycan hydrolase, endopeptidase, MepM, MepS, LytM domain, NlpC/P60 domain, MepH

## Abstract

Peptidoglycan (PG) is an essential component of the bacterial exoskeleton that plays a pivotal role in the maintenance of cell shape and resistance to cell lysis under high turgor pressures. The synthesis and degradation of PG must be tightly regulated during bacterial cell elongation and division. Unlike enzymes involved in PG synthesis, PG hydrolases show high redundancy in many bacteria including *Escherichia coli*. In this study, we showed that PG endopeptidases have distinct roles in cell growth and division. Phenotypic analysis of mutants lacking one of seven PG endopeptidases identified a MepM-specific phenotype, salt sensitivity, and a MepS-specific phenotype, EDTA sensitivity. Complementation test in each phenotype showed that the phenotype of the *mepM* mutant was restored only by MepM, whereas the phenotype of the *mepS* mutant was restored by MepS or by overexpression of MepH, PbpG, or MepM. These distinct phenotypes depend on both the specific localizations and specific domains of MepM and MepS. Finally, using the identified phenotypes, we revealed that MepM and MepH were genetically associated with both penicillin-binding protein 1a (PBP1a) and PBP1b, whereas MepS and PbpG were genetically associated with only PBP1b. Notably, a defect in PBP1a or PBP1b phenocopied the *mepM* mutant, suggesting the importance of MepM on PG synthesis. Therefore, our results indicate that each PG endopeptidase plays a distinct role in cell growth and division, depending on its distinct domains and cellular localizations.

## Introduction

Peptidoglycan (PG) is a macromolecule that forms a rigid mesh-like exoskeleton, which is required for shape maintenance and protection of bacteria from harsh environmental stresses, such as turgor pressure (Vollmer and Bertsche, 2008). PG is structurally composed of linear glycan strands cross-linked by short-peptide chains. The glycan strands are made of alternating β-1,4-linked sugars, N-acetylglucosamine (GlcNAc) and N-acetylmuramic acid (MurNAc), and a short-peptide chain composed of 2–5-amino-acid residues is covalently attached to the D-lactoyl moiety of each MurNAc. In *Escherichia coli*, the peptide chain is composed of L-alanine, D-glutamic acid, *meso*-diaminopimelic acid (*meso*-DAP), D-alanine, and D-alanine, and the cross-links between the neighboring peptide chains take place predominantly between the fourth D-alanine and the third *meso*-DAP or minorly between the third *meso*-DAP and the third *meso*-DAP (Glauner et al., 1988; Vollmer and Bertsche, 2008).

PG is not only a strong protective exoskeleton, but also a dynamic architecture that can be constantly expanded, degraded, and split during the growth and cell division (Vollmer and Bertsche, 2008). Generally, it is known that up to half of the preexisting PG is degraded and recycled (Dhar et al., 2018). To achieve the dynamic flexibility of PG, bacteria have many PG hydrolases, which can be classified into three groups, lytic transglycosylases, amidases, and peptidases (Vermassen et al., 2019). Lytic transglycosylases cleave the β-1,4-glycosidic bond between MurNAc and GlcNAc and catalyze the formation of an anhydro linkage between the C1 and C6 residues of MurNAc, which results in the formation of 1,6-anhydromuramic acid products (GlcNAc-anhydroMurNAc-peptide) (Holtje et al., 1975; Dhar et al., 2018). Amidases hydrolyze the lactylamide bond between MurNAc and the peptide chain; consequently, the cross-links of PG are broken (Uehara et al., 2010). Peptidases can be classified into two subgroups, endopeptidases that cleave within the cross-bridged peptide chains and carboxypeptidases that remove the C-terminal amino acid of the peptide chains (Vollmer et al., 2008; Vermassen et al., 2019). Additionally, based on the two isomeric forms of the cleaved amino acids, peptidases can be divided into DD, DL, and LD peptidases (Vollmer et al., 2008).

Bacteria have many PG hydrolases, and their functional redundancy has been reported (Vollmer et al., 2008; Vermassen et al., 2019). The functional consequence of this redundancy remains unclear. In several studies, specific functions of PG hydrolases have been revealed (Nambu et al., 1999; Peters et al., 2016; Santin and Cascales, 2017; Murphy et al., 2019). For example, FlgJ is a lytic transglycosylase that specifically functions to form a hole in the PG that is necessary for late flagella assembly (Nambu et al., 1999). Similarly, the lytic transglycosylase MltE is involved in the late stages of a type VI secretion system assembly (Santin and Cascales, 2017). Among various DD-carboxypeptidases, DacD (also known as PBP6b) is a specialized DD-carboxypeptidase that is more active at low pH and seems to function in cell shape maintenance in acidic environment (Peters et al., 2016).

In *E. coli*, there are seven proteins having DD-endopeptidase activity, MepA, MepH, MepM, MepS, AmpH, DacB, and PbpG (Vermassen et al., 2019). *In vitro* experiments have shown that MepA, MepH, MepM, MepS, and PbpG have only endopeptidase activity, whereas AmpH and DacB have carboxypeptidase and endopeptidase activities (van Heijenoort, 2011; Vermassen et al., 2019). Although a previous study has shown that MepH, MepM, and MepS are associated with cross-link cleavage in PG synthesis (Singh et al., 2012), their distinct roles remain unclear.

In this study, we investigated the phenotypes of endopeptidase-deficient mutants and the roles of various domains of endopeptidases. The *mepM* mutant exhibited a strong sensitivity to salt stress, whereas the *mepS* mutant was highly sensitive to EDTA. These distinct phenotypes depended on both the specific localization and a specific domain of each endopeptidase. Using the phenotypes identified, we showed that PG endopeptidases differentially affect penicillin-binding protein 1a (PBP1a) and PBP1b. Therefore, these results suggest that PG endopeptidases play distinct physiological roles, depending on their localizations and specific domains.

## Materials and Methods

### Bacterial Strains, Plasmids, and Culture Conditions

All *E. coli* strains and plasmids used in this study are presented in Supplementary Table S1. All primers used in this study are presented in Supplementary Table S2. Luria–Bertani (LB) medium or M9 minimal medium containing the indicated carbon and nitrogen sources was used for the *E. coli* cell culture. In M9 minimal medium, ammonium ion (NH_4_^+^) was used as the nitrogen source unless otherwise mentioned. Ampicillin (100 μg/mL), kanamycin (50 μg/mL), tetracycline (10 μg/mL), and chloramphenicol (5 μg/mL) were used when necessary. The bacterial growth under diverse culture conditions was examined using a 10-fold serial dilution spotting assay. The cells of the indicated strains including the wild-type strain were serially 10-fold diluted from 10^8^ to 10^4^ cells/mL, and 2 μL of diluted samples were spotted onto indicated plates, including LB plates, LB plates containing 750 mM NaCl, 1 mM EDTA, or/and various concentrations of arabinose, and M9 minimal medium plates containing the indicated carbon and nitrogen sources. The plates were incubated at 37°C until colonies of the wild-type cells of 10^4^ cells/mL appeared. Photographs of the plates were taken with a digital camera EOS 100D (Canon Inc., Japan).

The deletion of *E. coli* genes was performed using the λ red recombinase as described previously with some modifications (Datsenko and Wanner, 2000; Choi and Lee, 2019). To exchange the entire or specific regions of the target genes with the FRT sequence containing the kanamycin-resistance gene, deletion cassettes were amplified from pKD13 using the primer sets listed in Supplementary Table S2. Purified deletion cassettes were electroporated into MG1655 cells harboring the pKD46 plasmid, and the deletion mutants were selected on LB plates with kanamycin at 37°C or 30°C. The deletion of genes was confirmed by polymerase chain reaction (PCR) using other primer sets (see Supplementary Table S2) located in the outside of the replaced sequences. The kanamycin-resistance gene was removed by using a pCP20 plasmid expressing the FLP recombinase (Datsenko and Wanner, 2000). In order to minimize the effect on bacterial physiology, the pCP20 plasmid was cured at 37°C, but not at 42°C. The curing of the pCP20 plasmid was confirmed through the growth inhibition in LB medium containing ampicillin.

The pBAD24-based vectors for the expression of PG endopeptidases were constructed by using primer sequences (see Supplementary Table S2) covering the open reading frames of PG endopeptidase genes: forward primers possessing a 16 bp sequence overlapped with pBAD24 for recombination (5’-CTAGCAGGAGGAATTC-3’) and reverse primers with a 16 bp sequence overlapped with pBAD24 for recombination (5’-GCAGGTCGACTCTAGA-3’). The PCR product was inserted into the pBAD24 plasmid digested by *Eco*RI and *Xba*I, through the recombination between overlapped sequences using Infusion cloning (Clontech, United States). The recombinant plasmids were confirmed through sequence analysis. To construct the pBAD24-based vectors for the expression of MepM-Flag or MepS-Flag, the sequence encoding Flag was inserted into the pBAD24 plasmid digested by *Pst*I, through the recombination between overlapped sequences, which generates the plasmid pBAD-Flag. The *mepM* and *mepS* genes were cloned into the plasmid pBAD-Flag, which generate the plasmids pBAD-MepM-Flag and pBAD-MepS-Flag, respectively.

To construct the pBAD24-based vectors for the expression of chimeric PG endopeptidases with the transmembrane domain of MepM, the transmembrane domain (1–40 amino acids) of MepM was cloned into the plasmid pBAD-Flag, which resulted in the plasmid pBAD-MepM(N)-Flag. PG endopeptidases without the signal sequence were cloned into the plasmid pBAD-MepM(N)-Flag. Similarly, the N-terminal sequence (1–27 amino acids) of MepS that contains the signal sequence and the cysteine residue for palmitoylation was cloned into the plasmid pBAD-Flag, which generates the plasmid pBAD-MepS(N)-Flag, and then PG endopeptidases without the signal sequence were cloned into the plasmid pBAD-MepS(N)-Flag. To construct the plasmid pBAD-MepM(ΔC)-Flag, MepM without the C-terminal sequence between 408 and 440 amino acids was cloned into the plasmid pBAD-Flag. Similarly, to construct the plasmid pBAD-MepS(ΔC)-Flag, MepS without the C-terminal sequence between 184 and 188 amino acids was cloned into the plasmid pBAD-Flag. To construct the MepM and MepS chimeric proteins with the signal sequence of DsbA, the signal sequence of DsbA (1–19 amino acids) was cloned into the plasmid pBAD-Flag, which generates the plasmid pBAD-DsbA(N)-Flag. Next, MepM and MepS without the signal sequence were cloned into the plasmid pBAD-DsbA(N)-Flag, which generates pBAD-DsbA(N)-MepM-Flag and pBAD-DsbA(N)-MepS-Flag, respectively. The domain-swapped proteins were constructed by PCR using primers listed in Supplementary Table S2 and recombination using infusion cloning. The point mutations of one or two amino acids, such as MepM(H314A), MepS(C94A), and MepS(S28D&R29D), were constructed by PCR using the plasmids expressing the wild-type proteins as a template and *Dpn*I-dependent digestion of the template plasmids.

To construct the strains with chromosomal *mepM* or *mepS* gene under the arabinose promoter, the region including the *mepM* or *mepS* gene and the chloramphenicol resistance gene was amplified by PCR using pBAD-MepM or pBAD-MepS as a template, respectively. The PCR product was integrated into the neutral region between the *ygcE* and *queE* genes through the λ red recombinase. Similarly, to construct the strain with chromosomal *mepM* gene fused with a sequence coding for the 3 × Flag epitope at its 3’ end, the region including the *3* × *Flag* gene and the chloramphenicol resistance gene was amplified by PCR using pBAD-MepM-Flag as a template. The PCR product was integrated into the 3’ end of the *mepM* gene through the Λ red recombinase. The *mepM mepS* double mutant was constructed by the introduction of the *mepS* deletion in the *mepM* mutant. The *mepM mepS* double mutant was selected on an M9 minimal medium containing 0.2% glucose and 0.2% casamino acid at 37°C.

### Detection of Intracellular Levels of PG Endopeptidases

To determine the intracellular levels of chimeric or domain-swapped PG endopeptidases, we used monoclonal antibody against Flag-tag (Santa Cruz Biotechnology, United States). Cells were grown in LB medium containing 0.0001% arabinose to midlogarithmic phase, and 1 mL of cell culture was collected. After boiling for 5 min, the samples were analyzed with 4–20% sodium dodecyl sulfate (SDS)–polyacrylamide gradient gels. Immunoblotting was performed according to standard procedures using the anti-Flag antibody. To know whether MepM is a membrane protein, the strain expressing MepM-3 × Flag at its natural chromosomal locus was cultured in LB medium to midlogarithmic phase. The cell cultures (100 mL) were disrupted through a French pressure cell at 12,000 psi through two passages. After ultracentrifugation at 150,000 *g* for 120 min at 4°C, the soluble and membrane fraction were divided. Immunoblotting was performed according to standard procedures using anti-EIIA^*Ntr*^ (Lee et al., 2014), anti-PBP1a (Cusabio, China), and anti-Flag antibodies.

### Zymogram Assay for PG Endopeptidase Activity

The activity of PG endopeptidases was measured by zymogram assay as previously described with some modifications (Vaz and Filipe, 2015). The crude cell walls from *E. coli* cells were prepared from *E. coli* cultures (1 L) at the exponential phase of growth in LB medium (OD_600_ = 1) at 37°C. The cells were harvested by centrifugation at 7,000 rpm for 15 min at 4°C, and the cell pellet was washed with 200 mL of distilled water (DW). Cells were resuspended in 30 mL of DW and autoclaved for 15 min at 121°C. The crude cell walls were harvested by centrifugation at 12,000 rpm for 15 min at 4°C and stored at −20°C. The crude cell walls were thawed and resuspended in DW to 50 mg/mL final concentration. To make the polyacrylamide gel for the zymogram assay, 15 mg of the crude cell walls were used as a substrate in a 12% SDS–polyacrylamide gel electrophoresis gel. Purified PG endopeptidases resuspended in Laemmli loading buffer (100 mM Tris–HCl, pH 6.8, 17.2% glycerol, 0.02% bromophenol blue, and 0.2 M DTT) were loaded in the gel. After electrophoresis at 70 V, the gel was washed with 250 mL of DW thrice for 15 min to remove SDS and then incubated overnight in renaturation buffer (50 mM Tris–HCl, pH 7.5, 10 mM CaCl_2_, 10 mM MgCl_2_, and 0.1% Triton X-100) at 37°C with gentle shaking. The gel was stained in 250 mL of methylene blue solution (0.1% methylene blue in 0.01% KOH) for 1 h. The gel stained with methylene blue was destained in DW until a clear band indicating PG endopeptidase activity was observed in the opaque gel. Lysozyme was used as a positive control. NaCl was added to 925 mM in renaturation buffer if necessary.

### Quantitative Real-Time PCR

Total RNA was prepared using the RNeasy Mini Kit (Qiagen, United States) from wild-type cells in LB medium and the *mepS* mutant cells expressing PG endopeptidases in LB medium containing 1% arabinose grown to OD_600_ = 0.8, according to the manufacturer’s instructions. Genomic DNA of each sample was removed through the treatment with RNase-free DNase I (Promega, United States) at 37°C for at least 1 h. Approximately 1.2 μg of each extracted RNA was converted into cDNA using cDNA EcoDry Premix (Clontech, United States). Quantitative real-time PCR was performed using 10-fold diluted cDNAs as template, primers specific for PG endopeptidases or 16S rRNA, and 2X SYBR Premix Ex Taq II (Takara, Japan) in a reaction volume of 20 M L in triplicate. PCR and detection of amplified DNA products were performed using the CFX96 Real-Time System (Bio-Rad, United States). The relative expression level was calculated as the difference between the threshold cycle of the target gene and the threshold cycle of the reference gene (16S rRNA) for each sample.

## Results

### Distinct Phenotypes of PG Endopeptidase-Depleted Mutants

**FIGURE 1:**
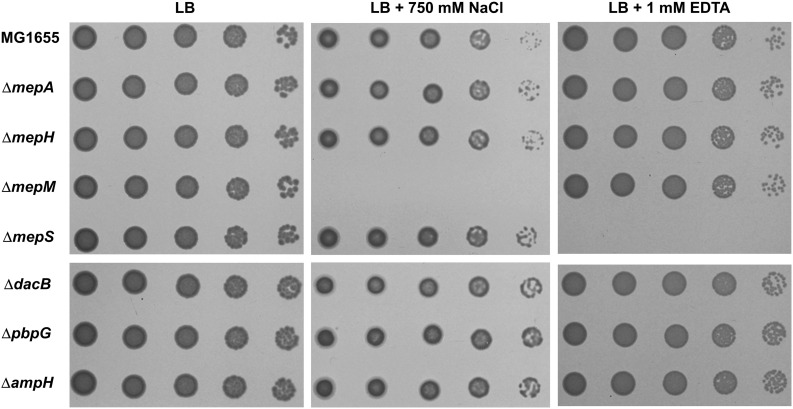
Distinct phenotypes of PG endopeptidase mutants. The wild-type and PG endopeptidase mutant cells were serially diluted from 10^8^ to 10^4^ cells/mL in 10-fold steps and spotted onto an LB plate or LB plates containing 750 mM NaCl or 1 mM EDTA as indicated.

To analyze the physiological roles of redundant PG endopeptidases, we constructed deletion mutants of all proteins with PG endopeptidase activity and examined the bacterial growth under various stress conditions, including envelope, oxidative, ethanol, EDTA, and carbon/nitrogen starvation stresses (Supplementary Figure S1). These experiments revealed that the growth of the *mepM* mutant was completely inhibited under salt stress, whereas the growth of the *mepS* mutant was completely inhibited under EDTA stress (Figure 1). These phenotypes were detected in liquid culture, and the morphological alterations under stress conditions were detected (Supplementary Figure S2). Notably, the growths of the *mepS* and *mepM* mutants were hardly affected under salt and EDTA stresses, respectively, indicating that these mutants have mutually exclusive phenotypes. The cells lacking other PG endopeptidases showed normal bacterial growth under these stress conditions. These completely exclusive phenotypes among PG endopeptidases prompted us to investigate how the distinct roles are allocated between PG endopeptidases.

### The Importance of the PG Endopeptidase Activity on Salt- and EDTA-Sensitive Phenotypes

To confirm that the PG endopeptidase activities of MepM and MepS are associated with these phenotypes, we constructed expression vectors for MepM and MepS mutant proteins that are deficient in PG endopeptidase activities. MepM is one of the LytM (lysostaphin) domain-containing proteins (EnvC, MepM, NlpD, and YgeR) (Uehara et al., 2009), and the LytM domain involved in the PG endopeptidase activity is located in the C terminus of MepM (Supplementary Figure S3). The ectopic expression of MepM using the pBAD plasmid with an arabinose-inducible promoter restored the growth of the *mepM* mutant under salt stress, but the expression of MepM lacking the LytM domain did not (Supplementary Figure S4A). MepM is a metalloendopeptidase requiring Zn^2+^ for its PG endopeptidase activity (Singh et al., 2012). We constructed the MepM(H314A) mutant in which the histidine residue required for the Zn^2+^ coordination was substituted to alanine. Having no PG endopeptidase activity (Supplementary Figure S5), MepM(H314A) did not restore the growth of the *mepM* mutant under salt stress (Supplementary Figure S4A), despite its expression level comparable to that of the wild-type MepM (Supplementary Figure S6), indicating that the PG endopeptidase activity of MepM is associated with the salt-sensitive phenotype. MepS (also called Spr) belonging to the NlpC/P60 peptidase superfamily has a conserved Cys(94)-His(145)-His(157) catalytic triad, and MepS(C94A) mutant has no PG endopeptidase activity (Singh et al., 2012). Expectedly, in contrast to wild-type MepS protein, the MepS(C94A) mutant protein did not restore the growth of the *mepS* mutant in the presence of 1 mM EDTA (Supplementary Figure S4B). Thus, these results show that the PG endopeptidase activity is associated with these phenotypes.

### Non-redundancy Within the PG Endopeptidase Family

**FIGURE 2:**
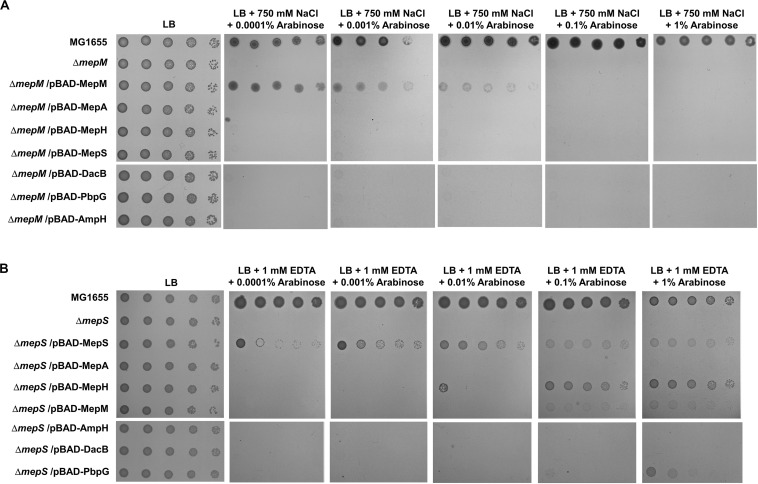
Complementation analysis of the phenotypes of the *mepM* and *mepS* mutants. **(A)** The wild-type, *mepM* mutant, and *mepM* mutant cells harboring the indicated plasmids were serially diluted from 10^8^ to 10^4^ cells/mL in 10-fold steps and spotted onto an LB plate or LB plates containing 750 mM NaCl and the indicated concentrations of arabinose. **(B)** The wild-type, *mepS* mutant, and *mepS* mutant cells harboring the indicated plasmids were serially diluted from 10^8^ to 10^4^ cells/mL in 10-fold steps and spotted onto an LB plate or LB plates containing 1 mM EDTA and the indicated concentrations of arabinose.

*E. coli* has seven proteins with PG endopeptidase activity, and MepS, MepM, and MepH show functional redundancy (Singh et al., 2012). To examine whether this redundancy can be applied to salt- and EDTA-sensitive phenotypes, we performed complementation analysis using the pBAD plasmid. Salt sensitivity of the *mepM* mutant could be complemented only by MepM, and other PG endopeptidases including MepS hardly affected this phenotype at any arabinose concentration tested (Figure 2A). These results suggest a specific role of MepM. EDTA sensitivity of the *mepS* mutant was complemented only by MepS at low arabinose concentrations, but at high arabinose concentrations, other PG endopeptidases, including MepH, PbpG, and MepM, could complement this phenotype (Figure 2B and Supplementary Figure S7). These results indicate that MepS also has a distinct role under EDTA stress, but its role can be replaced by overproduction of several PG endopeptidases, such as MepH, PbpG, and MepM (Supplementary Figure S8). Taken together, these results suggest that each PG endopeptidase plays a distinct role.

### The Importance of the Localization of MepM and MepS for Their Functions

**FIGURE 3:**
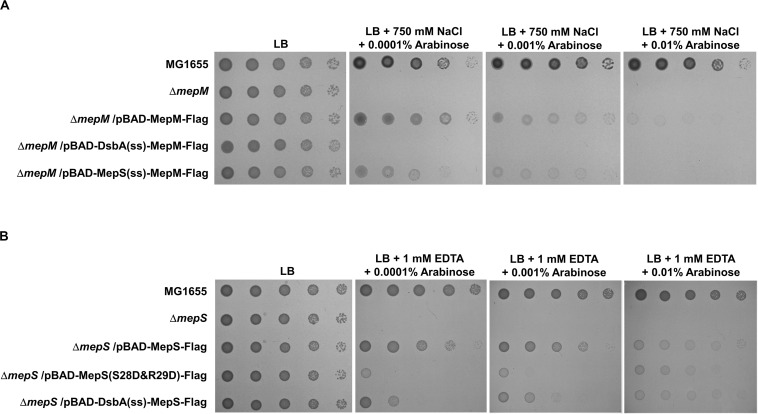
The importance of the localization of MepM and MepS on their function. **(A)** The wild-type, *mepM* mutant, and *mepM* mutant cells harboring the indicated plasmids were serially diluted from 10^8^ to 10^4^ cells/mL in 10-fold steps and spotted onto an LB plate or LB plates containing 750 mM NaCl and the indicated concentrations of arabinose. **(B)** The wild-type, *mepS* mutant, and *mepS* mutant cells harboring the indicated plasmids were serially diluted from 10^8^ to 10^4^ cells/mL in 10-fold steps and spotted onto an LB plate or LB plates containing 1 mM EDTA and the indicated concentrations of arabinose.

Based on the signal sequence predictions of PG endopeptidases, they are classified into three groups: the inner membrane (IM) protein MepM, the outer membrane (OM) lipoprotein MepS, and the periplasmic soluble proteins (Supplementary Figure S3). The N-terminus of MepM is predicted to be a transmembrane domain, and the membrane localization of MepM was confirmed by Western blot analysis (Supplementary Figure S9). Because MepM and MepS show a distinct localization, we analyzed the role of the localization in the MepM- and MepS-specific phenotypes. We constructed three chimeric proteins as follows: MepM-Flag with the Flag-tag at the C-terminus, DsbA(ss)-MepM-Flag with both the signal sequence of the periplasmic protein DsbA at the N-terminus (Paradis-Bleau et al., 2010; Tsang et al., 2017) instead of the transmembrane domain of MepM and the Flag-tag at the C-terminus to direct it to the periplasm, and MepS(ss)-MepM-Flag with both the signal sequence and the palmitoylation residue of MepS at the N-terminus and the Flag-tag at the C-terminus to target it to the OM. Complementation experiments using these proteins showed that MepM-Flag restored the growth of the *mepM* mutant under the high salt condition and MepS(ss)-MepM-Flag restored growth only partially, whereas DsbA(ss)-MepM-Flag did not restore growth (Figure 3A). These results indicate the importance of the IM localization for the full activity of MepM. Similarly, we constructed three chimeric proteins as follows: MepS-Flag with the Flag-tag at the C-terminus, DsbA(ss)-MepS-Flag with the signal sequence of DsbA at the N-terminus instead of its own signal sequence and the palmitoylation residue to target it to the periplasm, and MepS(S28D&R29D)-Flag whose OM-targeted signal (Ser^2+^-Arg^3+^) is substituted to the IM retention signal (Asp^2+^-Asp^3+^) (Grabowicz, 2018). Like MepM, mislocalized MepS proteins also have the partial activity, but they could not sufficiently restore the growth of the *mepS* mutant under EDTA stress at low arabinose concentrations (Figure 3B). Western blot analysis using an anti–Flag-tag antibody showed that mislocalized MepM and MepS proteins were sufficiently expressed in the cells (Supplementary Figure S10). In summary, these results suggest that the accurate localization of MepM and MepS is required for their full activity.

### The Roles of MepM and MepS Are Inimitable by Other PG Endopeptidases

**FIGURE 4:**
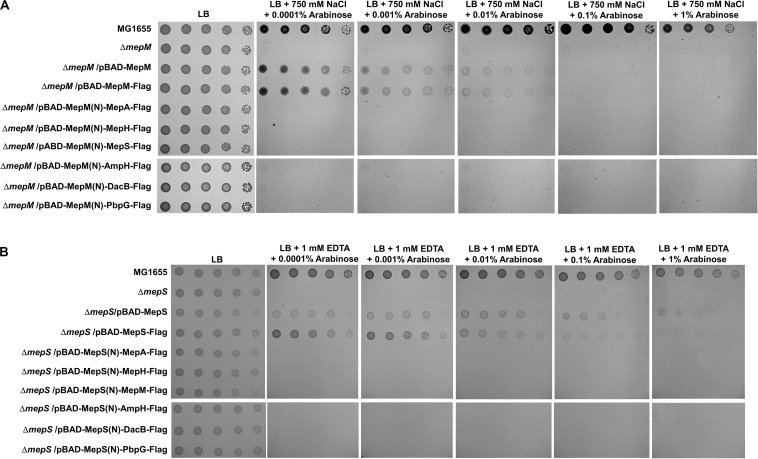
Distinct roles of MepM and MepS are not due to their unique localization. **(A)** The wild-type, *mepM* mutant, and *mepM* mutant cells harboring the indicated plasmids were serially diluted from 10^8^ to 10^4^ cells/mL in 10-fold steps and spotted onto an LB plate or LB plates containing 750 mM NaCl and the indicated concentrations of arabinose. **(B)** The wild-type, *mepS* mutant, and *mepS* mutant cells harboring the indicated plasmids were serially diluted from 10^8^ to 10^4^ cells/mL in 10-fold steps and spotted onto an LB plate or LB plates containing 1 mM EDTA and the indicated concentrations of arabinose.

Because the unique localization of MepM and MepS is important for their functions, we wonder whether their distinct roles are due to their unique localizations. To address this issue, we constructed chimeric PG endopeptidases localized in the IM or OM, through the replacement of each N-terminal sequence with the MepM transmembrane domain or MepS N-terminus sequence containing the palmitoylation residue and OM-targeted signal residues (Ser^2+^-Arg^3+^), respectively. Each chimeric protein contains the Flag-tag at the C-terminus. Although MepM containing Flag-tag restored the growth of the *mepM* mutant in high salt almost up to the level comparable to that of the wild-type MepM, the other PG endopeptidases including MepS hardly complemented the phenotype of the *mepM* mutant (Figure 4A). Similar results were obtained regarding MepS (Figure 4B). Western blot analysis using an anti–Flag-tag antibody showed that all chimeric proteins were sufficiently expressed in the cells (Supplementary Figure S11). These results strongly suggest that the distinct roles of MepM and MepS are inimitable by targeting other PG endopeptidases to the IM and OM, respectively. It is noteworthy that overexpressed chimeric MepH, PbpG, and MepM proteins did not restore the growth defect of the *mepS* mutant (Figure 4B), although overexpression of MepH, PbpG, and MepM in their native forms could complement the growth defect of the *mepS* mutant at least in part (Figure 2B). These results indicate that MepH and PbpG also require accurate localization (periplasmic localization) for their functions.

### The Entire Domain of MepM and MepS Is Responsible for Their Function

To identify the domain(s) responsible for the MepM-specific function, we constructed domain-swapped proteins between MepM and MepS or NlpD as follows: the chimeric NlpD protein with the transmembrane and LytM domains of MepM and the chimeric MepM protein with the NlpC/P60 domain of MepS (Supplementary Figure S12A). MepM has relatively long amino acid sequences downstream of the LytM domain compared to other LytM proteins (Supplementary Figure S13). Because the removal of this amino acid sequence almost completely abolished the function of MepM (Supplementary Figure S12B), we constructed domain-swapped proteins containing this sequence (Supplementary Figure S12A). Although the Western blot showed the adequate expression of the chimeric proteins (Supplementary Figure S14), they hardly complemented the phenotype of the *mepM* mutant (Supplementary Figure S12B). These results indicate that both the LytM domain and other regions of MepM are important for its function.

Among PG endopeptidases, only MepS and MepH belong to the NlpC/P60 peptidase superfamily (Supplementary Figure S3). To identify the domain(s) related to the MepS-specific function, we constructed domain-swapped proteins between MepS and MepH (Supplementary Figure S15A). Because, like MepM, the removal of the amino acid sequence downstream of the NlpC/P60 domain significantly abolished the function of MepS (Supplementary Figure S15B), despite the adequate expression (Supplementary Figure S16), we constructed domain-swapped proteins containing this amino acid sequence. Both MepS with the NlpC/P60 domain of MepH and MepH with the NlpC/P60 domain of MepS hardly restored the growth of the *mepS* mutant (Supplementary Figure S15B), also suggesting the importance of both the NlpC/P60 domain and other regions for the specific function of MepS. In summary, these results show that the distinct roles of MepM and MepS are due to their different amino acid sequences, as well as their distinct localization.

### Distinct Effects of MepM, MepS, MepH, and PbpG on PBP1a and PBP1b

**FIGURE 5:**
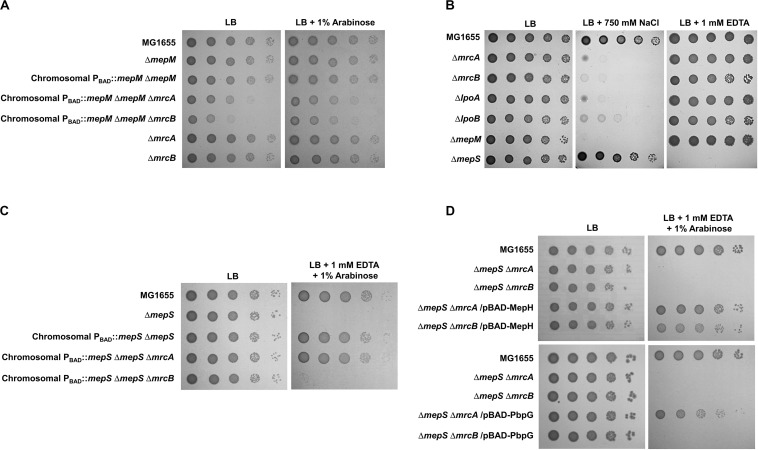
Distinct effects of MepM, MepS, MepH, and PbpG on PBP1a and PBP1b. **(A)** The effect of MepM on PBP1a and PBP1b. The cells of the indicated strains were serially diluted from 10^8^ to 10^4^ cells/mL in 10-fold steps and spotted onto an LB plate or an LB plate containing 1% arabinose. **(B)** Salt sensitivity of the *mrcA*, *mrcB*, *lpoA*, and *lpoB* mutants. The cells of the indicated strains were serially diluted from 10^8^ to 10^4^ cells/mL in 10-fold steps and spotted onto an LB plate or LB plates containing 750 mM NaCl or 1 mM EDTA as indicated. **(C)** The effect of MepS on PBP1b. The cells of the indicated strains were serially diluted from 10^8^ to 10^4^ cells/mL in 10-fold steps and spotted onto an LB plate or an LB plate containing 1 mM EDTA and 1% arabinose. **(D)** The effects of MepH and PbpG on PBP1a and PBP1b. The cells of the indicated strains were serially diluted from 10^8^ to 10^4^ cells/mL in 10-fold steps and spotted onto an LB plate or an LB plate containing 1 mM EDTA and 1% arabinose.

PG endopeptidases have been predicted to function as space makers that cleave the cross-links for insertion of newly synthesized PG strands (Singh et al., 2012; Lai et al., 2017). PG synthesis in *E. coli* is performed by PBP-containing complexes. Among them, PBP1a (encoded by an *mrcA* gene) and PBP1b (encoded by an *mrcB* gene) are class A PBP proteins that possess both glycosyltransferase and transpeptidase activities. They are not essential for survival, but the *mrcA mrcB* double mutant is not viable (Paradis-Bleau et al., 2010; Typas et al., 2010). To examine whether MepM is genetically associated with PBP1a and PBP1b, we constructed *mrcA mepM* and *mrcB mepM* double mutants and tested their salt sensitivity. Both *mrcA mepM* and *mrcB mepM* double mutants showed the growth defect even under normal growth conditions (Figure 5A), indicating that MepM is genetically associated with both PBP1a and PBP1b and may play a major role in PG synthesis. Notably, through the salt-sensitivity experiment, we found that, like the *mepM* mutant, both the *mrcA* and *mrcB* mutants were also significantly sensitive to 750 mM NaCl (Figure 5B). The same phenotype was observed in mutants defective for the lipoprotein cofactors LpoA and LpoB that are essential for the functions of PBP1a and PBP1b, respectively (Paradis-Bleau et al., 2010; Typas et al., 2010). These growth defects were restored by ectopic expression of each protein (Supplementary Figure S17). Although several PBP1b-related phenotypes, such as β-lactam susceptibility (Schmidt et al., 1981; Paradis-Bleau et al., 2010), were observed, an apparent PBP1a-related phenotype has not been identified yet. To our knowledge, we are the first to show an apparent phenotype of the *mrcA* mutant. Our results demonstrate that a defect in PG synthesis causes high sensitivity to salt stress.

Similarly, we constructed the *mrcA mepS* and *mrcB mepS* double mutants. Unlike MepM, both mutants did not show any growth defect under normal growth conditions (Figure 5C), indicating that the effect of MepS in PG synthesis is weaker than that of MepM. Interestingly, under EDTA stress, the expression of MepS could complement the growth defect of the *mepS* mutant only in the presence of PBP1b (Figure 5C). These results imply that MepS is genetically associated with only PBP1b. Because the phenotype of the *mepS* mutant was also complemented by the overproduction of MepH and PbpG (Figure 2B), we performed similar experiments using MepH and PbpG. Overproduced MepH complemented the growth defect of the *mepS* mutant both in the absence of PBP1a and PBP1b, whereas overproduced PbpG complemented the growth defect of the *mepS* mutant only in the presence of PBP1b (Figure 5D). Therefore, these results strongly imply that MepM and MepH are genetically associated with both PBP1a and PBP1b, whereas MepS and PbpG are genetically associated with only PBP1b.

### The Importance of MepM in Adaptation to Salt Stress

**FIGURE 6:**
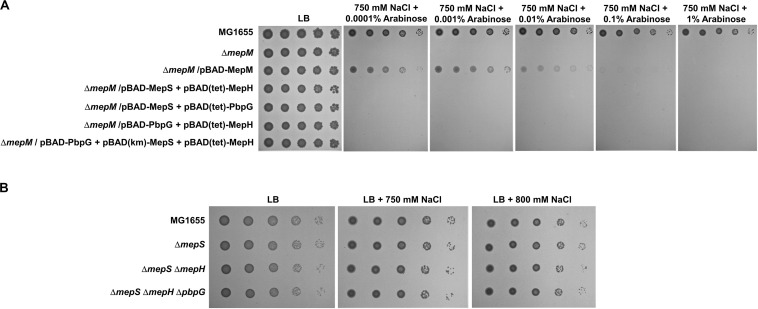
The importance of MepM in adaptation to salt stress. **(A)** The effect of simultaneous overexpression of MepS, MepH, and PbpG on salt sensitivity of the *mepM* mutant. The cells of the wild-type, *mepM* mutant, and *mepM* mutant harboring the indicated plasmids were serially diluted from 10^8^ to 10^4^ cells/mL in 10-fold steps and spotted onto an LB plate or LB plates containing 750 mM NaCl and the indicated concentrations of arabinose. **(B)** The effect of the *mepS mepH* double or *mepS mepH pbpG* triple deletion on adaptation to salt stress. The wild-type and the indicated mutant cells were serially diluted from 10^8^ to 10^4^ cells/mL in 10-fold steps and spotted onto an LB plate or LB plates containing 750 or 800 mM NaCl as indicated.

We observed salt sensitivity in the *mrcA*, *mrcB*, *lpoA*, and *lpoB* mutants (Figure 5B). These results strongly suggest that the full activity of PG synthesis is required for adaptation to osmotic stress. Among PG endopeptidases, only the *mepM* mutant was strongly sensitive to salt stress (Figure 1). Although MepH and MepM were genetically associated with both PBP1a and PBP1b (Figure 5), overexpression of MepH did not complement the salt sensitivity of the *mepM* mutant (Figure 2A). Because, in addition to MepM and MepH, MepS, and PbpG also affected PG synthesis (Figure 5), we examined whether co-overexpression of MepH, MepS, and PbpG complements the phenotype of the *mepM* mutant. No combination of overexpression of three proteins restored salt sensitivity of the *mepM* mutant (Figure 6A), despite their proper expressions (Supplementary Figure S18). These results imply that MepM plays a major role in PG synthesis. This is also supported by the following experiment. Because a severe defect of PG synthesis, such as a defect of PBP1a or PBP1b, resulted in salt sensitivity, we wondered whether the *mepS mepH* double or *mepS mepH pbpG* triple mutant is sensitive to salt stress. Expectedly, neither mutant was sensitive to salt stress (Figure 6B). Taken together, our results indicate that MepM alone is sufficient for adaptation to salt stress, which implies its major role in PG synthesis.

## Discussion

PG is a pivotal macromolecule of bacterial exoskeleton that is necessary for maintaining cell shape and overcoming osmotic stress in most bacteria (Vollmer and Bertsche, 2008), and several proteins involved in its synthesis are targets of diverse antibiotics, including β-lactams (Zapun et al., 2008). Bacteria have diverse PG hydrolases responsible for the synthesis and regulation of cell wall (Vermassen et al., 2019). In *E. coli*, cytoplasmic enzymes involved in PG precursor synthesis show an approximately 1:1 stoichiometric ratio between enzymes and reactions (14 enzymes for 12 reactions), whereas periplasmic proteins involved in PG synthesis and degradation, such as PG hydrolases, show high redundancy (more than 36 enzymes for 9 reactions) (Pazos et al., 2017; Mueller et al., 2019). Recently, several reports have analyzed the physiological significance of PG hydrolase redundancy and revealed distinct roles for several PG hydrolases (Hugonnet et al., 2016; Peters et al., 2016; Schaub et al., 2016; Santin and Cascales, 2017; More et al., 2019). However, the physiological significance of the redundant PG endopeptidases was not in-depth analyzed. In this study, we revealed distinct roles among PG endopeptidases. MepM, MepS, MepH, and PbpG showed different phenotypes, different physiological significance, and differential effects on PBP1a and PBP1b.

In this study, we presented MepM- and MepS-specific phenotypes. The MepM- and MepS-specific phenotypes were restored only by expressing MepM and MepS, respectively (Figure 2). Overexpressed MepH, PbpG, and MepM suppressed the phenotype of the *mepS* mutant, but not the phenotype of the *mepM* mutant. A previous report has also shown that the growth defect of the *mepS* mutant on NA medium at high temperature was suppressed by MepS, MepM, and MepH (Singh et al., 2012). Because the study did not analyze the suppression pattern according to the expression levels, the authors concluded that the three PG endopeptidases were redundant. However, our results showed that only the *mepS* mutant was sensitive to EDTA stress, and this phenotype was suppressed only by MepS. Other PG endopeptidases could suppress the phenotype of the *mepS* mutant only when overproduced. Therefore, our results show the distinct role of MepS. The salt sensitivity of the *mepM* mutant was complemented by MepM only at low arabinose concentrations (Figure 2A). This may be partly caused by a toxic effect of overexpression of MepM (Supplementary Figure S19). Notably, a similar pattern was also found in the salt sensitivity of the *mrcA* and *mrcB* mutants (Supplementary Figure S17). Weak complementation by LpoA or LpoB was observed at 0.1 and 1% arabinose concentrations. These results imply that the tight regulation of MepM, PBP1a, and PBP1b activities is necessary for adaptation to salt stress.

MepM seems to be a major PG endopeptidase in *E. coli*. The salt sensitivity of the *mepM* mutant was not suppressed by the overexpression of other PG endopeptidases, alone or simultaneously (Figures 2A, 6A). The growth of the *mepM mrcA* and *mepM mrcB* double mutant was inhibited even under normal growth conditions (Figure 5A). MepS also seems to be an important PG endopeptidase. The phenotype of the *mepS* mutant was restored only by MepS when not overproduced. A previous report has shown that the *mepM mepS* double mutant did not survive in LB medium (Singh et al., 2012). This was also confirmed by our results (Supplementary Figure S20). These results indicate that MepM and MepS are the main PG endopeptidases.

**FIGURE 7:**
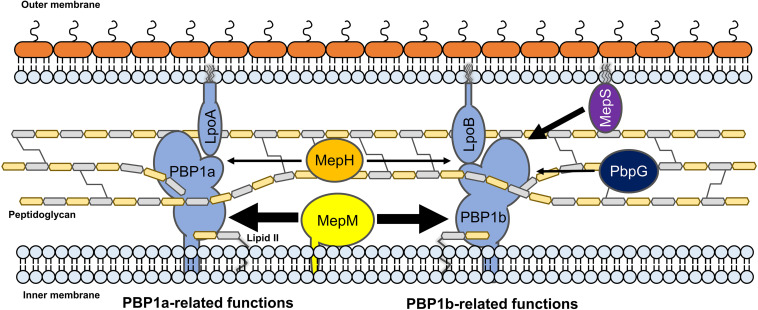
The model for the distinct roles of MepM, MepS, MepH, and PbpG. MepM and MepS are located in the inner membrane and outer membrane, respectively, whereas MepH and PbpG are located in the periplasm. MepM is a transmembrane protein, whereas MepS is a lipoprotein. MepM is genetically associated with both PBP1a and PBP1b, and MepH supports its function. MepS is genetically associated with PBP1b, and PbpG supports its function. The width of arrows indicates the degree of the effects of endopeptidases on PBP1a or PBP1b.

Several experiments using MepM- and MepS-specific phenotypes demonstrated that each PG endopeptidase was differentially associated with PBP1a and PBP1b. MepM was genetically associated with both PBP1a and PBP1b, whereas MepS was genetically associated with only PBP1b (Figures 5A,C). This difference was also found in MepH and PbpG; MepH was genetically associated with both PBP1a and PBP1b, whereas PbpG was genetically associated with only PBP1b (Figure 5D). Based on these results, a model regarding the roles of PG endopeptidases is presented in Figure 7. MepM is the major PG endopeptidase genetically related to both PBP1a and PBP1b, and MepH supports its role. MepS is the PBP1b-specific PG endopeptidase, and PbpG supports its role. It is noteworthy that overexpression of PbpG more efficiently complemented the phenotype of the *mepS* mutant under a condition where PBP1a is deleted than in the presence of PBP1a (Figures 2B, 5D). Although we do not know the exact reason for these results, there is a possibility that PBP1a inhibits the physiological role of PbpG. Further experiments are required to investigate the possibility.

Based on the signal sequence predictions of PG endopeptidases, they localize in different cellular sites as follows; MepM in the IM, MepS in the OM, and other PG endopeptidases in the periplasm. Notably, the functions of MepM, MepS, MepH, and PbpG are dependent on their localization; thus, mislocalization of these PG endopeptidases significantly reduced their activities (Figures 3, 4). Identification of partner proteins that function together with PG endopeptidases will explain these results. We also showed that the distinct roles of MepM and MepS are associated with the entire domain of each protein, and not only with the specific domains, such as the LytM, LysM, and NlpC/P60 domains (Supplementary Figures S12, S15), suggesting that the distinct role-related regions of MepM and MepS are distributed throughout the entire sequence of the proteins.

It is noteworthy that chimeric MepS with the Flag-tag at the C-terminus more efficiently complemented the growth defect of the *mepS* mutant than the wild-type MepS (Figure 4B). Prc, an ATP-independent periplasmic tail-specific protease, degrades MepS through the recognition of its C-terminal residues (Singh et al., 2015; Su et al., 2017), suggesting that the Flag-tag at the C-terminus of MepS could inhibit degradation by Prc. Therefore, increased stability of the chimeric MepS seems to result in more efficient complementation of the phenotype. Notably, we also found that the C-terminal residues of MepM and MepS are necessary for their functions (Supplementary Figures S12, S15). Therefore, further studies analyzing the roles of the C-terminal residues of MepM and MepS are required.

In this study, we used the specific phenotypes of the *mepM* and *mepS* mutants for analyzing the distinct roles of PG endopeptidases but did not demonstrate the exact physiological significance of these phenotypes. Only the *mepM* mutant among PG endopeptidases was strongly sensitive to salt stress (Figure 1). Notably, this phenotype was also detected in the *mrcA*, *mrcB*, *lpoA*, and *lpoB* mutants (Figure 5B). These results strongly suggest that the defect of either PBP1a or PBP1b does not affect the bacterial growth under normal growth conditions, but the full activity of PG synthesis is required for adaptation to osmotic stress. Therefore, the salt sensitivity of the *mepM* mutant may be due to severe defect in PG synthesis. Because the *mepM mepS* double mutant did not survive in LB medium (Supplementary Figure S20; Singh et al., 2012), the *mepM* mutant could be sensitive to salt if the enzymatic activity of MepS is strongly inhibited by salt. However, this hypothesis is not correct. The enzymatic activities of MepM and lysozyme were significantly inhibited by salt, whereas that of MepS was not affected by salt (Supplementary Figure S21). Therefore, salt sensitivity of the *mepM* mutant seems not to be caused by salt-mediated inhibition of the MepS activity. On the other hand, EDTA sensitivity of the *mepS* mutant seems to be caused by EDTA-mediated inhibition of the MepM activity. The *mepM mepS* double mutant was not viable in LB medium (Supplementary Figure S20; Singh et al., 2012), and the activity of the metalloendopeptidase MepM was inhibited in the presence of EDTA (Singh et al., 2012). Therefore, EDTA-mediated inhibition of MepM could be responsible for the lethality of the *mepS* mutant. This assumption was also supported by the fact that in the presence of EDTA the wild-type strain was sensitive to salt stress, like the *mepM* mutant (Supplementary Figure S22). These results imply that the addition of EDTA might inhibit the MepM activity. However, further experiments are required to examine the accuracy of this assumption.

PG endopeptidases have been predicted to function as space makers that trigger PG enlargement for insertion of a new glycan strand (Burman and Park, 1984; Singh et al., 2012; Lai et al., 2017). In this study, our results based on genetic analysis indirectly support this prediction. MepM and MepH seem to be genetically associated with both PBP1a and PBP1b, whereas MepS and PbpG seem to be genetically associated with PBP1b alone. Biochemical studies on the physical interactions between PBP1a or PBP1b and PG endopeptidases are required to confirm this model. Because all four PG endopeptidases were genetically associated with PBP1b, it is necessary to analyze the physiological significance of the presence of diverse PBP1b-related PG endopeptidases in further experiments.

## Data Availability Statement

All datasets presented in this study are included in the article/Supplementary Material.

## Author Contributions

C-RL contributed to the conception and the design of experiments. SP, YK, HL, Y-JS, and C-RL researched and wrote the manuscript. All authors contributed to the article and approved the submitted version.

## Conflict of Interest

The authors declare that the research was conducted in the absence of any commercial or financial relationships that could be construed as a potential conflict of interest.
