# Supplementary material for: Genetic Evidence for Distinct Functions of Peptidoglycan Endopeptidases in Escherichia coli
Source: Front Microbiol. 2020 Sep 11;11:565767. doi: 10.3389/fmicb.2020.565767 (PMC7516022; doi:10.3389/fmicb.2020.565767)
Supplement: Supplementary file 1 [file Table_1.docx]

Supplementary Material

# Supplementary Figures and Tables

## Supplementary Figures

**
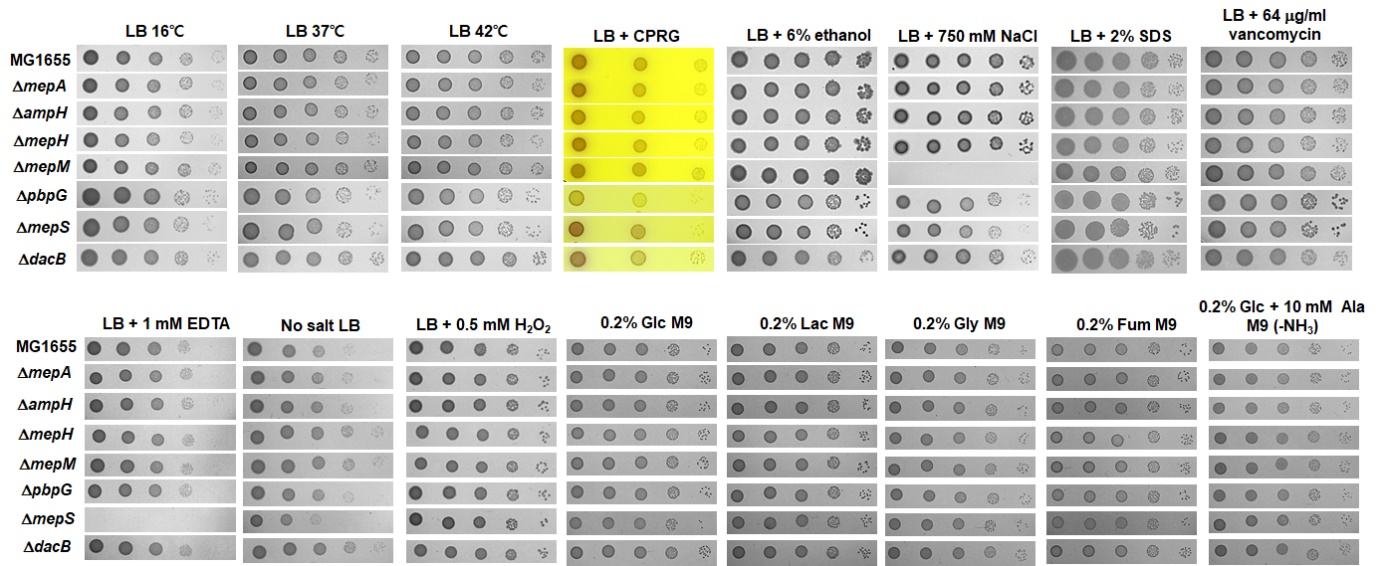
**

**Supplementary Figure 1.** Phenotype analysis of PG endopeptidase mutants. The cells of wild-type and PG endopeptidase mutant strains were serially diluted from 10^8^ to 10^4^ cells/ml in 10-fold steps and spotted onto LB plates, LB plates containing indicated materials, or M9 minimal medium plates containing indicated carbon and nitrogen sources. CPRG means the addition of 20 μg/ml of chlorophenyl red-β-d-galactopyranoside. No salt LB means LB medium without NaCl. M9 (-NH_3_) means M9 minimal medium without NH_3_. Plates were incubated at 37°C, if not otherwise indicated. Glc, glucose; Lac, lactose; Gly, glycerol; Fum, fumarate; Ala, alanine.


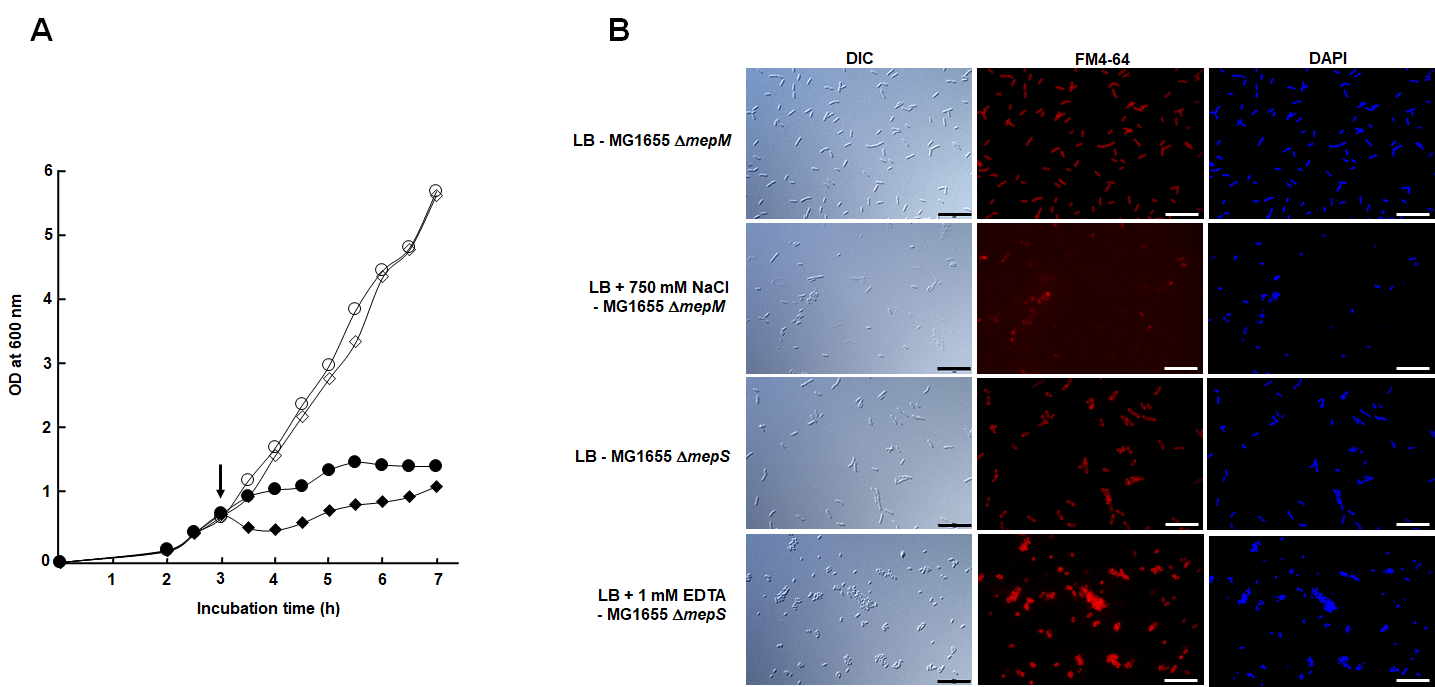


**Supplementary Figure 2.** Morphological change of the *mepM* and *mepS* mutant under stress conditions. **(A)** The *mepM* or *mepS* mutant cells grown in LB overnight were inoculated in LB medium. EDTA (1 mM) or NaCl (750 mM) was added in LB medium when OD_600nm_=0.8 (arrow). Cell growth was recorded by measuring the optical density at 600 nm: open diamonds, Δ*mepM* in LB medium without the addition of NaCl; closed diamonds, Δ*mepM* in LB medium with the addition of NaCl; open circles, Δ*mepS* in LB medium without the addition of EDTA; and closed circles, Δ*mepS* in LB medium with the addition of EDTA. **(B)** At 90 min after the addition of EDTA or NaCl, the *mepM* or *mepS* mutant cells grown in indicated medium were stained with FM4-64 (red) and DAPI (blue), and then spotted on a 1% agarose pad. Scale bars, 20 μm.


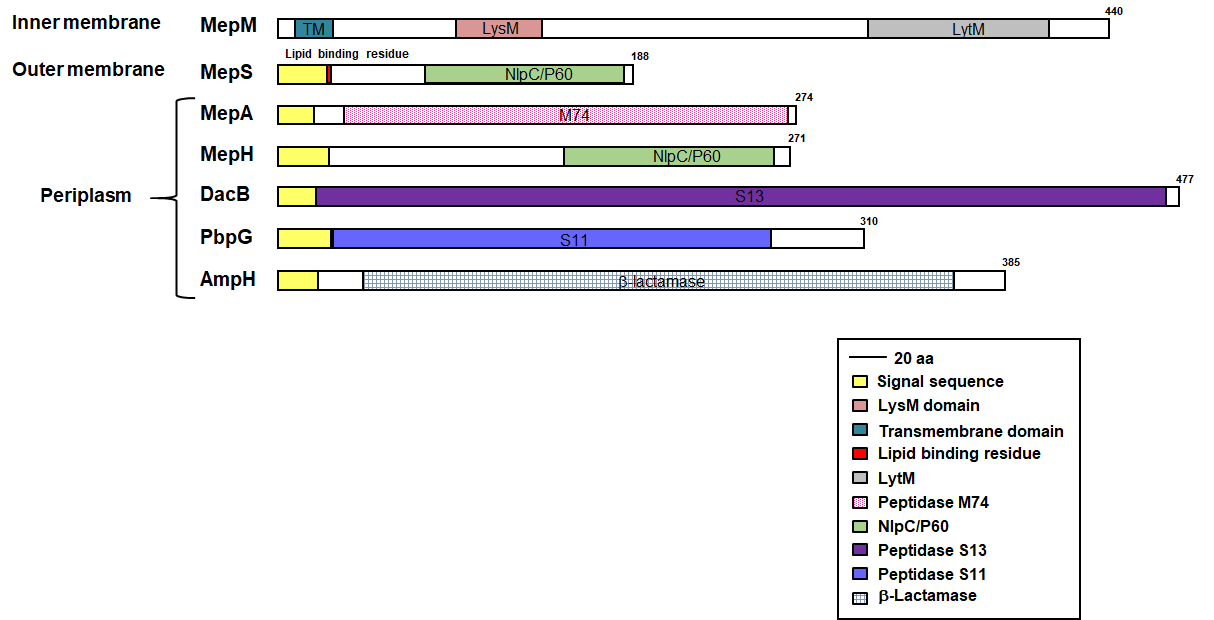


**Supplementary Figure 3.** Schematic representation of the domain organization of seven PG endopeptidases in *E. coli*.


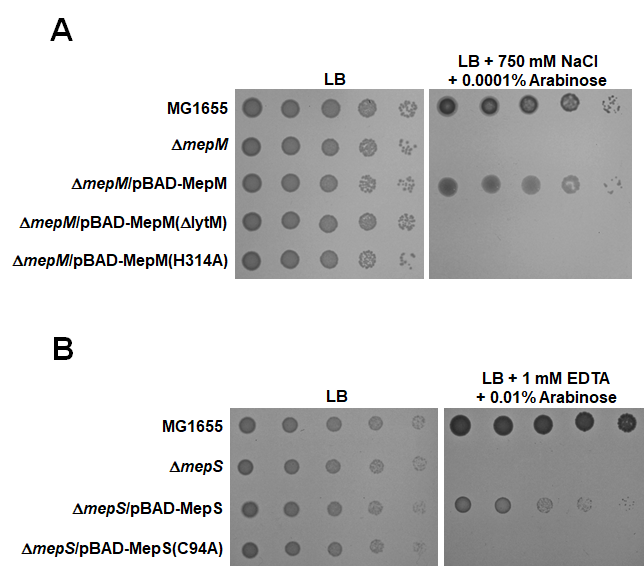


**Supplementary Figure 4.** The phenotypes of the *mepM* and *mepS* mutants are associated with their PG endopeptidase activity. **(A)** The wild-type, *mepM* mutant, and *mepM* mutant cells harboring indicated plasmids were serially diluted from 10^8^ to 10^4^ cells/ml in 10-fold steps and spotted onto an LB plate or an LB plate containing 750 mM NaCl and 0.0001% arabinose. **(B)** The wild-type, *mepS* mutant, and *mepS* mutant cells harboring indicated plasmids were serially diluted from 10^8^ to 10^4^ cells/ml in 10-fold steps and spotted onto an LB plate or an LB plate containing 1 mM EDTA and 0.01% arabinose.

**
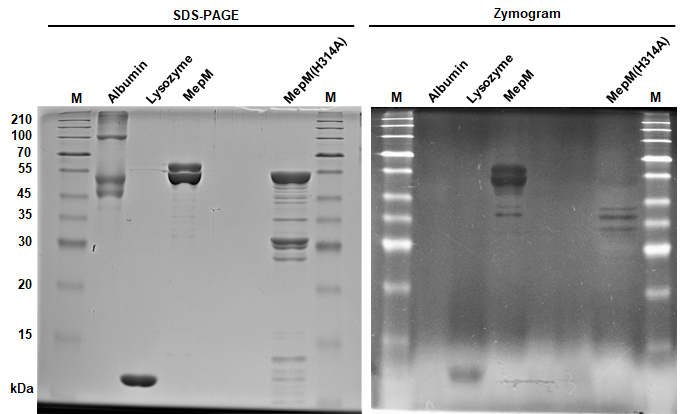
**

**Supplementary Figure 5.** Examination of PG endopeptidase activities of MepM and MepM(H314A). Purified proteins were separated in a 12% SDS PAGE gel containing the crude bacterial cell walls, and the washed gel was incubated overnight in renaturation buffer at 37°C. The gel was stained in methylene blue solution and destained in DW until a clear band with PG endopeptidase activity was observed in the opaque gel as described in Materials and Methods. The Coomassie blue-stained SDS-PAGE gel (left) and the zymogram assay gel (right) are shown. Lysozyme and albumin were used as positive and negative controls, respectively. M, prestained molecular weight markers (Koma Biotech, Korea).


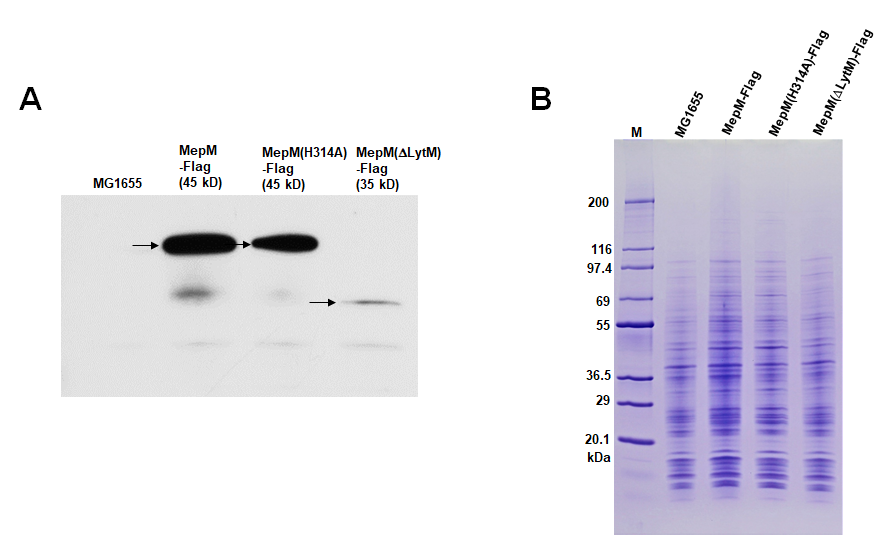


**Supplementary Figure 6.** Determination of the expression levels of MepM-Flag, MepM(H314A)-Flag, MepM(ΔLytM)-Flag. **(A)** The cells of the *mepM* mutants expressing indicated MepM proteins were grown in LB medium containing 0.0001% arabinose. Cells (2 x 10^8^ cells) were harvested at the exponential phase and western blot analysis using the anti-Flag antibody was performed to determine cellular levels of MepM proteins. The arrows indicate MepM proteins. **(B)** SDS-PAGE gels are presented as a loading control. M, Molecular weight markers.


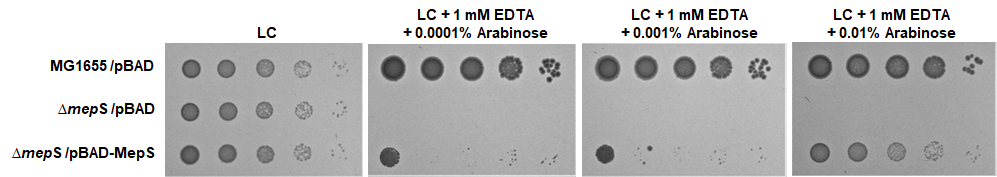


**Supplementary Figure 7.** Complementation of the phenotype of the *mepS* mutant. The wild-type harboring pBAD, *mepS* mutant harboring pBAD, and *mepS* mutant cells harboring pBAD-MepS were serially diluted from 10^8^ to 10^4^ cells/ml in 10-fold steps and spotted onto an LB plate with chloramphenicol (LC) or an LB plate containing chloramphenicol, 1 mM EDTA, and the indicated concentrations of arabinose.


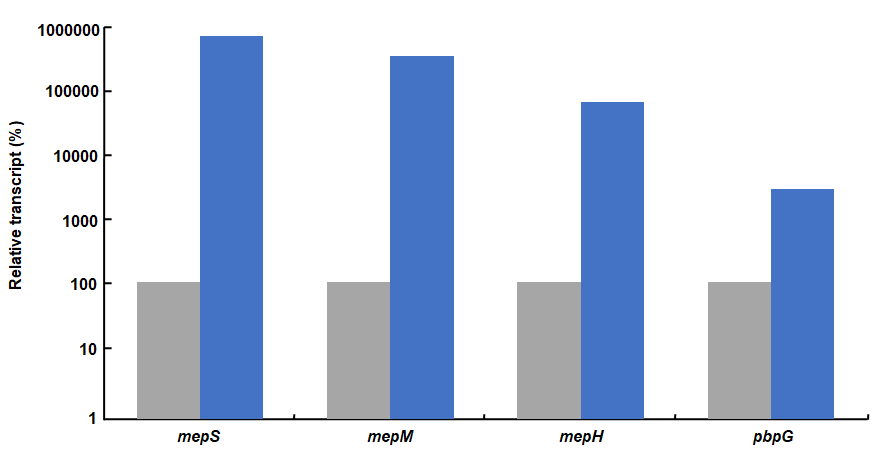


**Supplementary Figure 8.** Overexpression of PG endopeptidases in LB medium containing 1% arabinose. The total RNAs were extracted from the wild-type (gray bars) in LB medium and the *mepS* mutant strain expressing the indicated PG hydrolases (blue bars) in LB medium containing 1% arabinose at the exponential phase. Transcript levels were analyzed by qRT-PCR with primers specific for PG endopeptidases or 16S rRNA. The mRNA levels were normalized to the concentration of 16S rRNA.


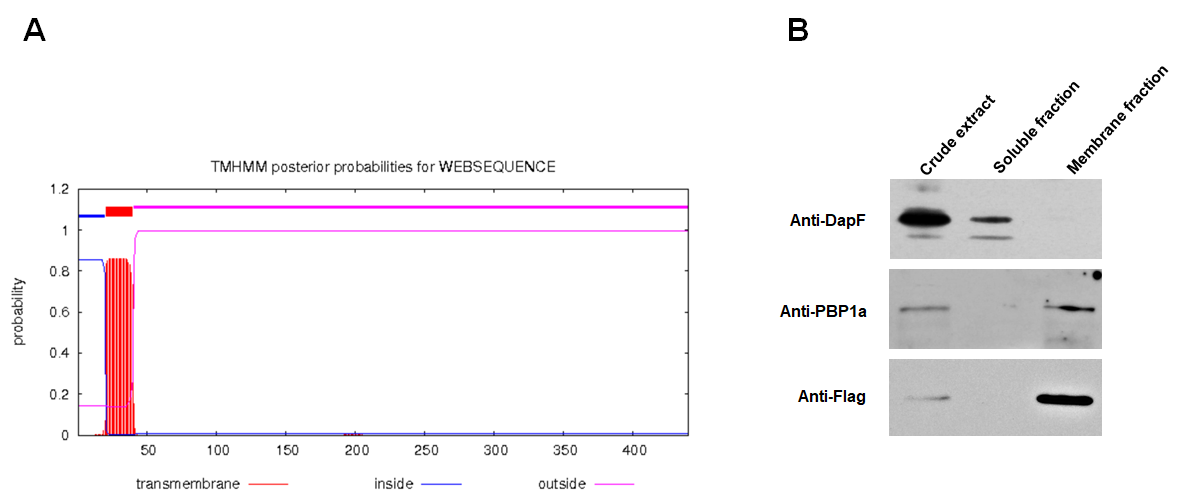


**Supplementary Figure 9.** MepM is a membrane protein. **(A)** The transmembrane domain of MepM is predicted by the TMHMM Server v. 2.0 program (http://www.cbs.dtu.dk/services/TMHMM/). **(B)** The MG1655 strain cells expressing chromosomal MepM-Flag were fractionated. DapF, PBP1a, MepM were detected using anti-DapF, anti-PBP1a, and anti-Flag antibodies, respectively.


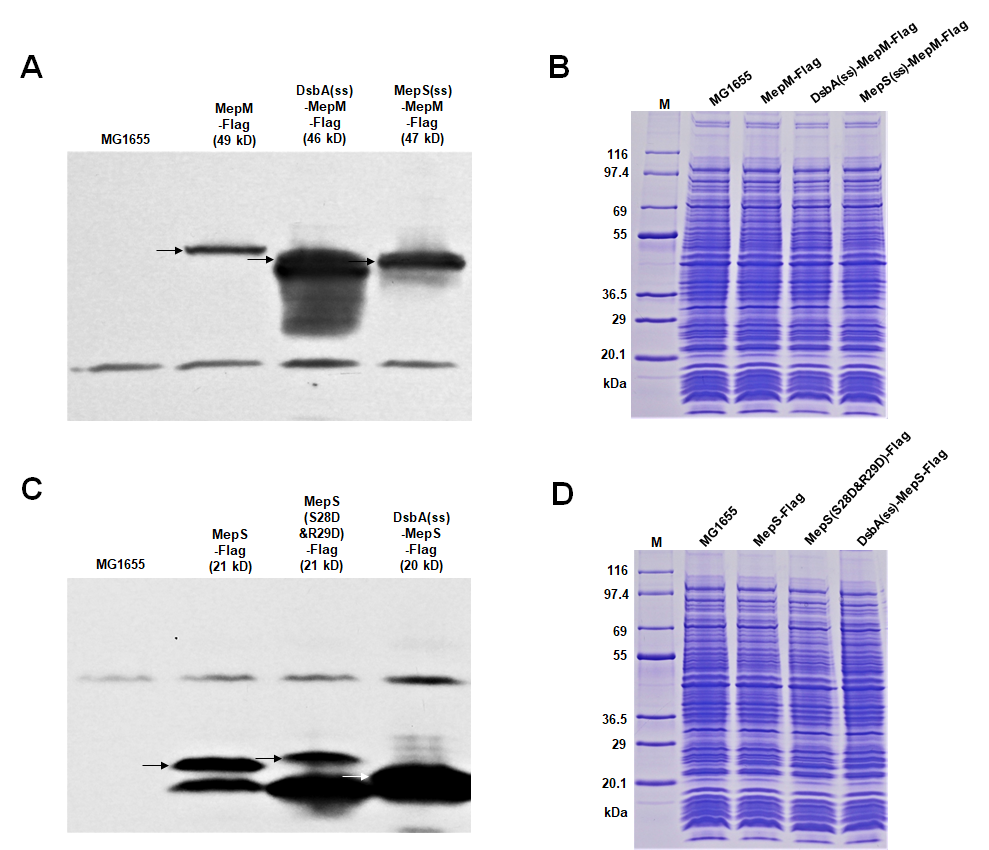


**Supplementary Figure 10.** Determination of the expression levels of Flag epitope-tagged MepM and MepS proteins. **(A and C)** The cells of the *mepM* **(A)** and *mepS* **(C)** mutants expressing indicated MepM or MepS proteins were grown in LB medium containing 0.0001% arabinose. Cells (4 x 10^8^) were harvested at the exponential phase and western blot analysis using the anti-Flag antibody was performed to determine cellular levels of indicated proteins. The arrows indicate MepM or MepS proteins. **(B and D)** SDS-PAGE gels are presented as loading controls for **(A)** and **(C)**, respectively. M, Molecular weight markers.


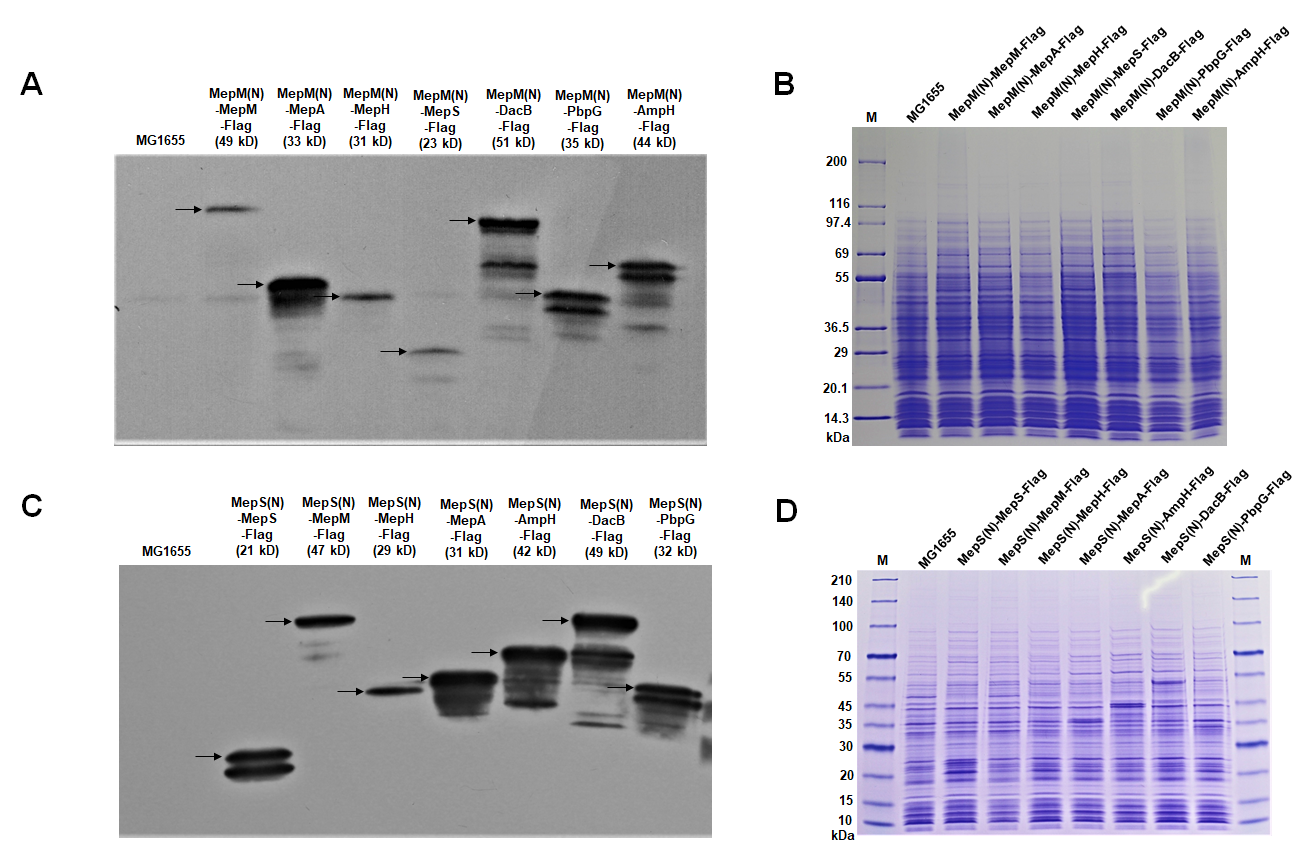


**Supplementary Figure 11.** Determination of the expression levels of Flag epitope-tagged chimeric PG endopeptidases. **(A and C)** The cells of the *mepM* **(A)** and *mepS* **(C)** mutants expressing indicated chimeric PG endopeptidases were grown in LB medium containing 0.0001% arabinose. Cells (4 x 10^8^ cells for the *mepM* mutant and 2 x 10^8^ cells for the *mepS* mutant) were harvested at the exponential phase and western blot analysis using the anti-Flag antibody was performed to determine cellular levels of chimeric PG endopeptidases. The arrows indicate chimeric PG endopeptidases. **(B and D)** SDS-PAGE gels are presented as loading controls for **(A)** and **(C)**, respectively. M, Molecular weight markers.


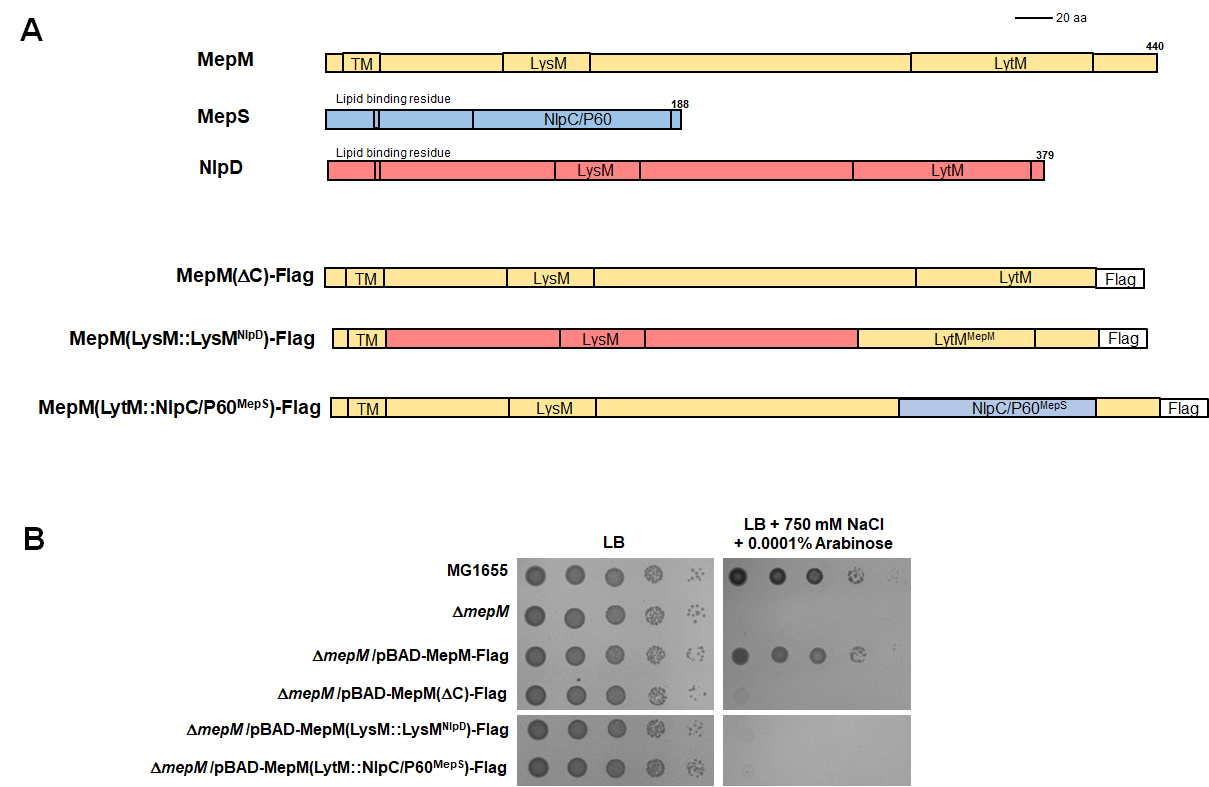


**Supplementary Figure 12.** Complementation analysis of the phenotypes of the *mepM* mutant by domain-swapped proteins. **(A)** Schematic representation of the domain organization of domain-swapped or C-terminus-deleted MepM proteins in *E. coli*. **(B)** The wild-type, *mepM* mutant, and *mepM* mutant cells harboring the indicated plasmids were serially diluted from 10^8^ to 10^4^ cells/ml in 10-fold steps and spotted onto an LB plate or LB plates containing 750 mM NaCl and 0.0001% arabinose.

**
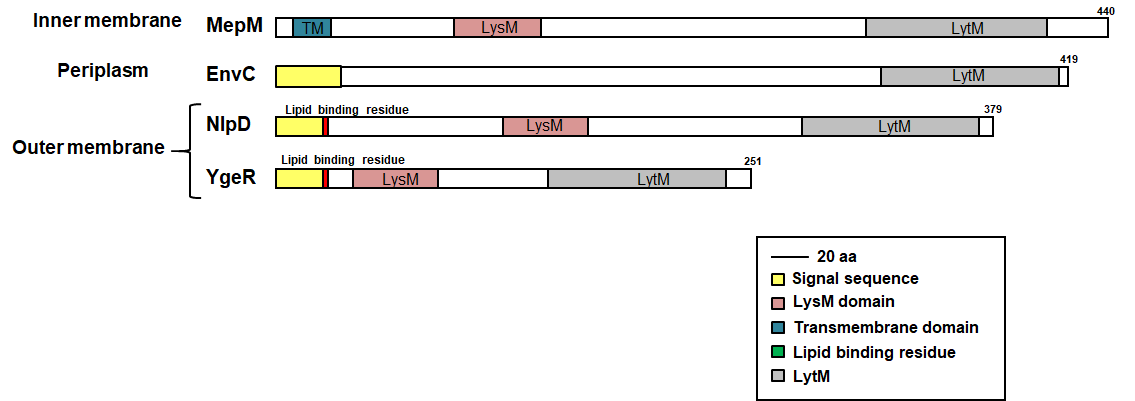
**

**Supplementary Figure 13.** Schematic representation of the domain organization of four LytM domain-containing proteins in *E. coli*.

**
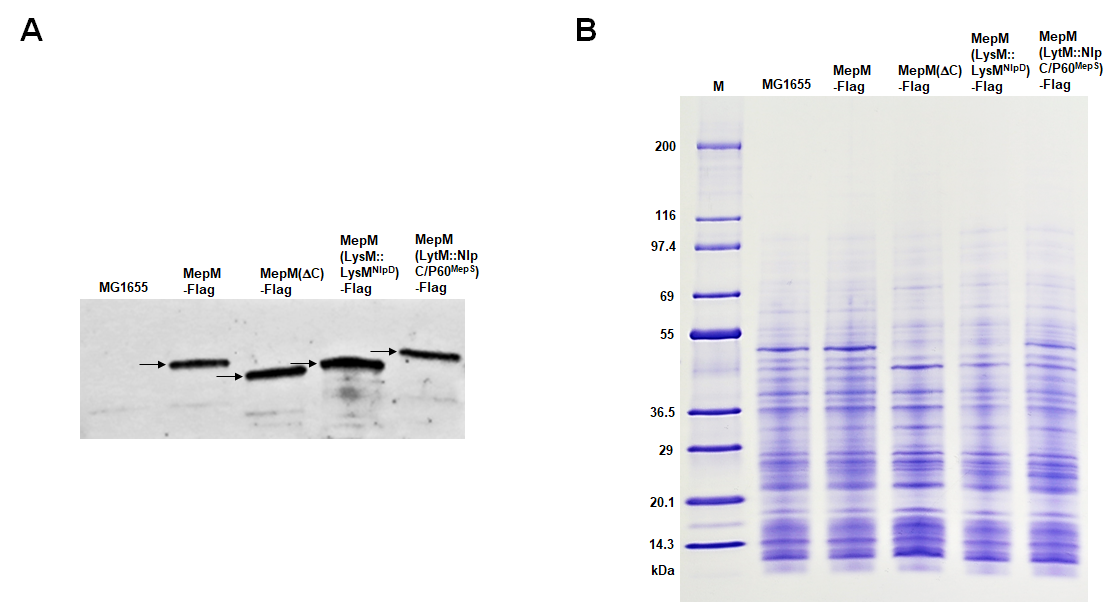
**

**Supplementary Figure 14.** Determination of the expression levels of domain-swapped or C-terminus-deleted Flag epitope-tagged MepM proteins. The cells of the wild-type and *mepM* mutant expressing indicated domain-swapped or C-terminus-deleted MepM proteins were grown in LB medium containing 0.0001% arabinose. Cells (2 x 10^8^ cells) were harvested at the exponential phase and western blot analysis using the anti-Flag antibody was performed to determine intracellular levels of domain-swapped MepM proteins. The arrows indicate chimeric PG endopeptidases. The SDS-PAGE gel was presented as a loading control. M, molecular weight markers.


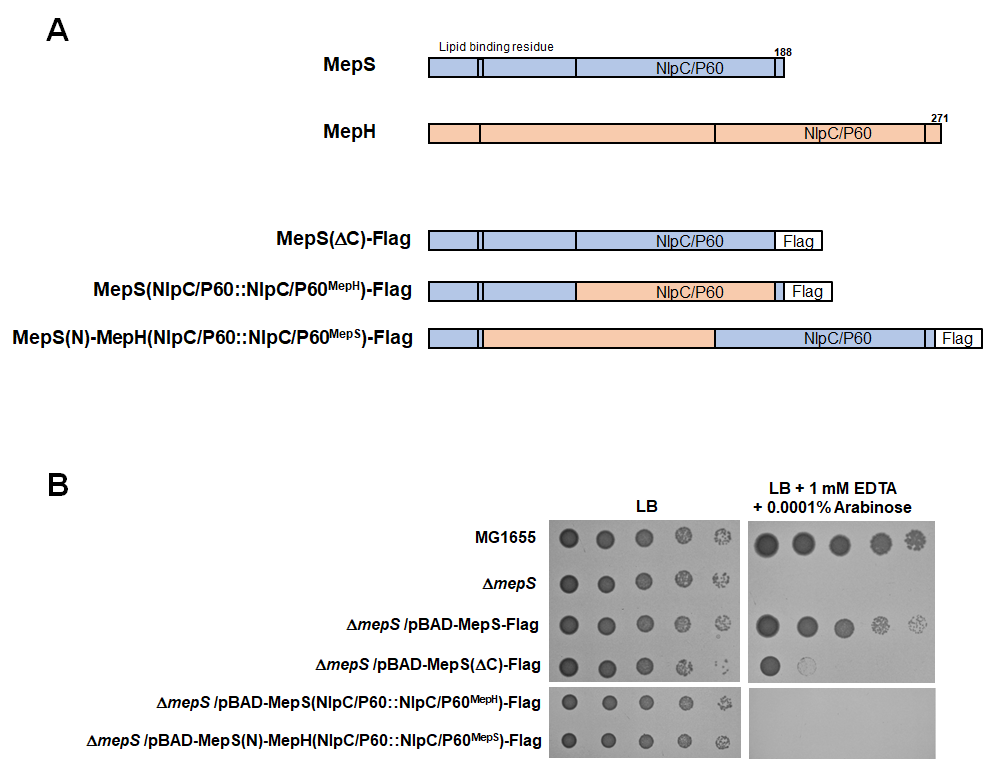


**Supplementary Figure 15.** Complementation analysis of the phenotypes of the *mepS* mutant by domain-swapped proteins. **(A)** Schematic representation of the domain organization of domain-swapped or C-terminus-deleted MepS proteins in *E. coli*. **(B)** The wild-type, *mepS* mutant, and *mepS* mutant cells harboring the indicated plasmids were serially diluted from 10^8^ to 10^4^ cells/ml in 10-fold steps and spotted onto an LB plate or LB plates containing 1 mM EDTA and 0.0001% arabinose.

**
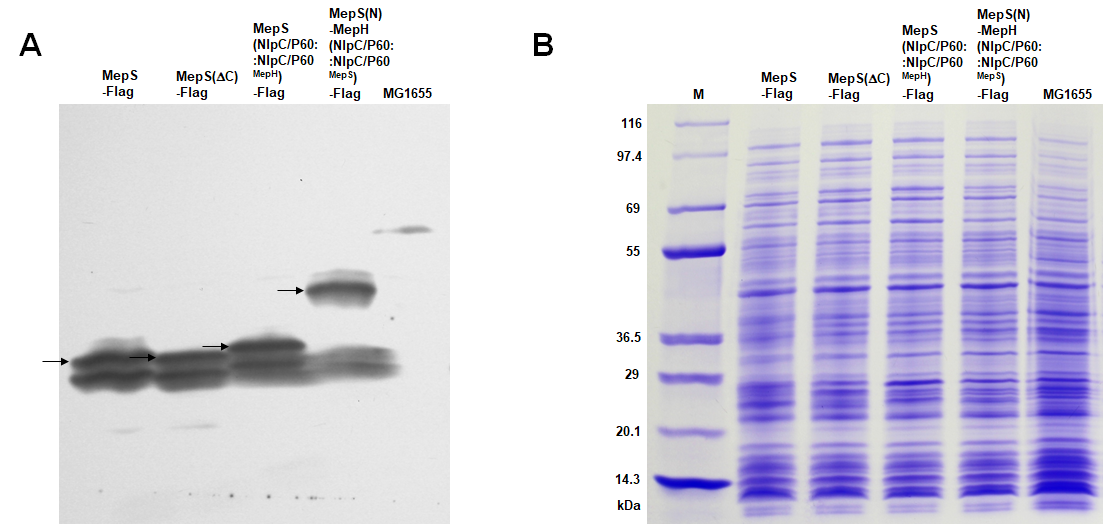
**

**Supplementary Figure 16.** Determination of the expression levels of domain-swapped or C-terminus-deleted Flag epitope-tagged MepS proteins. **(A)** The cells of the wild-type and *mepS* mutant expressing indicated domain-swapped or C-terminus-deleted MepS proteins were grown in LB medium containing 0.0001% arabinose. Cells (2 x 10^8^ cells) were harvested at the exponential phase and western blot analysis using the anti-Flag antibody was performed to determine intracellular levels of domain-swapped MepS proteins. The arrows indicate chimeric PG endopeptidases. **(B)** The SDS-PAGE gel was presented as a loading control. M, molecular weight markers.


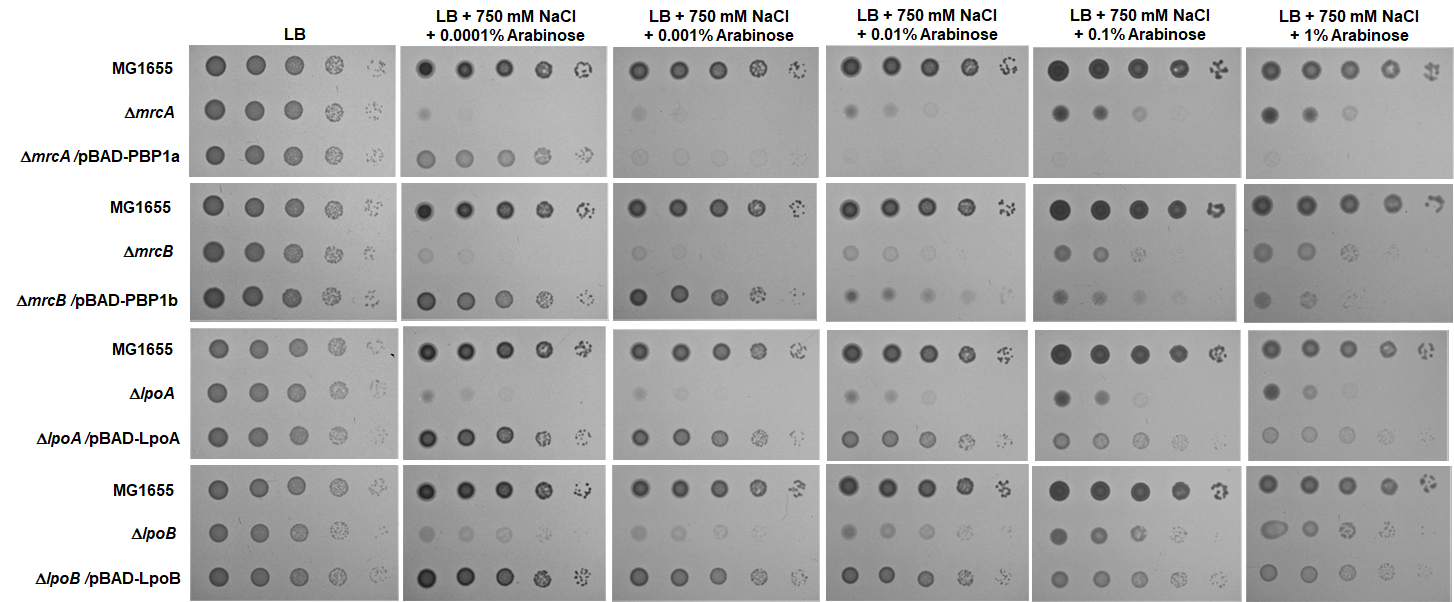


**Supplementary Figure 17.** Complementation of the salt sensitivity phenotype of the *mrcA*, *mrcB*, *lpoA*, and *lpoB* mutants. The cells of indicated strains were serially diluted from 10^8^ to 10^4^ cells/ml in 10-fold steps and spotted onto an LB plate or LB plates containing 750 mM NaCl and indicated concentrations of arabinose.


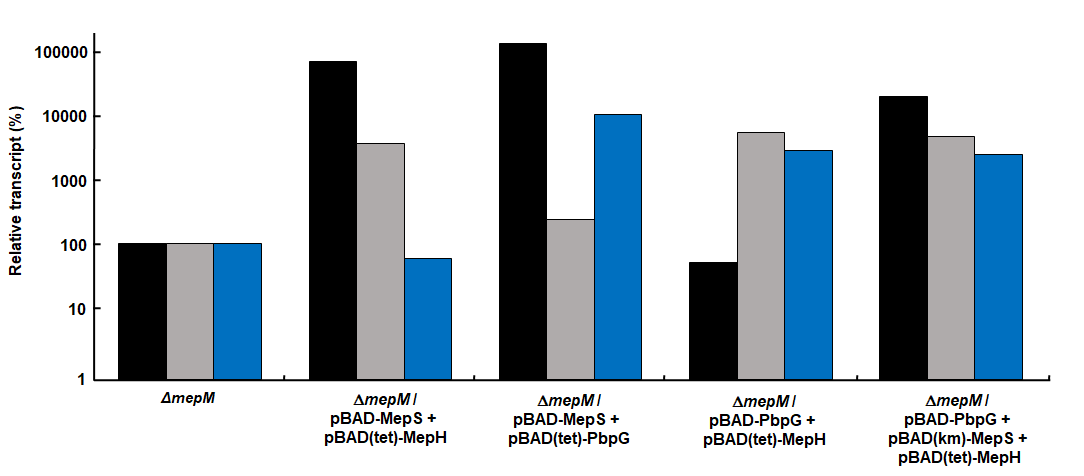


**Supplementary Figure 18.** Overexpression of PG endopeptidases in LB medium containing 0.1% arabinose. The total RNAs were extracted from the *mepM* mutant and the *mepM* mutant strain expressing the indicated PG hydrolases in LB medium containing 0.1% arabinose at the exponential phase. Transcript levels were analyzed by qRT-PCR with primers specific for PG endopeptidases or 16S rRNA. The mRNA levels were normalized to the concentration of 16S rRNA. Black bars, *mepS*; gray bars, *mepH*; blue bars, *pbpG*.


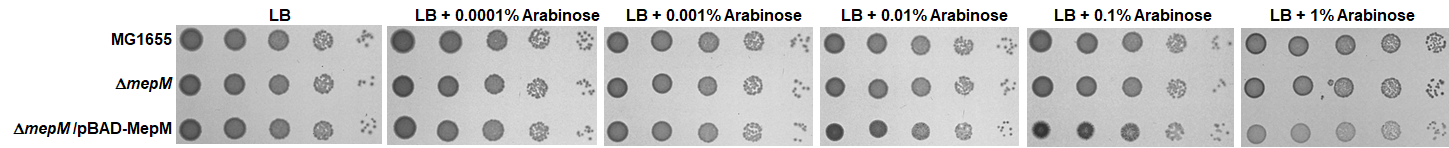


**Supplementary Figure 19.** Growth retardation of MepM overexpressing cells. The wild-type, *mepM* mutant, and *mepM* mutant cells harboring pBAD-MepM were serially diluted from 10^8^ to 10^4^ cells/ml in 10-fold steps and spotted onto an LB plate or LB plates containing the indicated concentrations of arabinose.

**
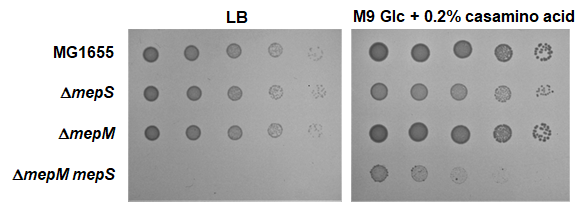
**

**Supplementary Figure 20.** The lethality of the *mepM mepS* double mutant in LB medium. The cells of wild-type, *mepM*, *mepS*, and *mepM mepS* double mutant strains were serially diluted from 10^8^ to 10^4^ cells/ml in 10-fold steps and spotted onto an LB plate or a M9 minimal medium plate containing 0.2% glucose and 0.2% casamino acid.

**
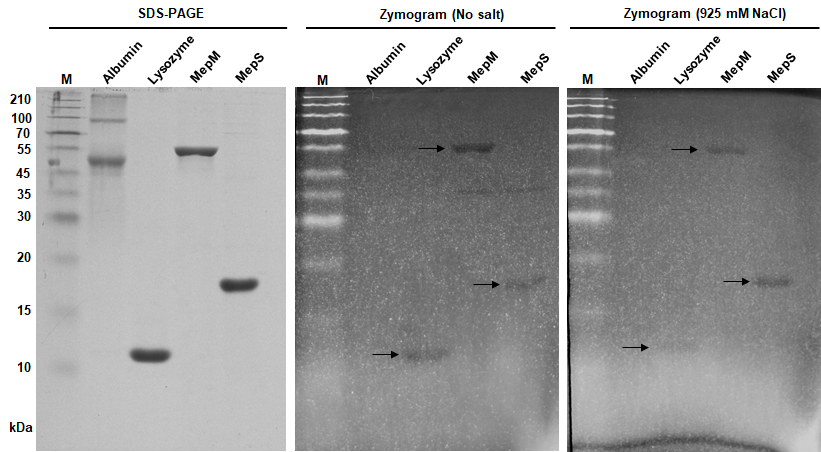
**

**Supplementary Figure 21.** The effect of salt on PG endopeptidase activities of MepM and MepS. Purified proteins MepM and MepS were separated in 10% non-SDS PAGE gel containing boiled substrate cells, and the washed gel was incubated overnight at 37°C in renaturation buffer with or without 925 mM NaCl. The gel was staining in methylene blue solution and destained in DW until a clear band with PG endopeptidase activity was observed in the opaque gel as described in Materials and Methods. Lysozyme and albumin were used as positive and negative controls, respectively. M, prestained molecular weight markers (Koma Biotech, Korea).

**
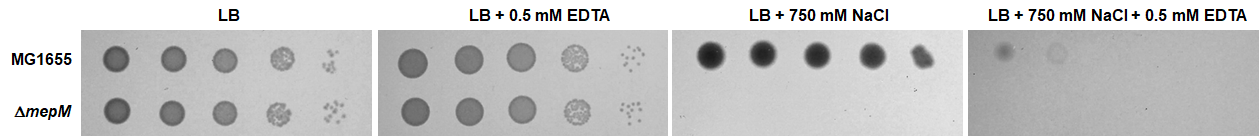
**

**Supplementary Figure 22.** The addition of EDTA phenocopies the *mepM* mutant. The wild-type and *mepM* mutant cells were serially diluted from 10^8^ to 10^4^ cells/ml in 10-fold steps and spotted onto an LB plate or LB plates containing 0.5 mM EDTA, 750 mM NaCl, or both (0.5 mM EDTA and 750 mM NaCl).

## Supplementary Tables

**Supplementary Table S1.** ***Escherichia coli* strains and plasmids used in this study.**

| Strain or plasmid | Genotype or phenotype | Source or Reference |
| --- | --- | --- |
| **Strains** |  |  |
| MG1655 | F^-^ λ^-^ *ilvG*^-^ *rfb*-50 *rph*-1. Wild type *E. coli* K-12 | (Blattner et al., 1997) |
| MG1655Δ*mepA* | MG1655 *mepA:: frt* | This study |
| MG1655Δ*mepH* | MG1655 *mepH*:: *frt* | This study |
| MG1655Δ*mepM* | MG1655 *mepM*:: *frt* | This study |
| MG1655Δ*mepS* | MG1655 *mepS*:: *frt* | This study |
| MG1655Δ*dacB* | MG1655 *dacB*:: *frt* | This study |
| MG1655Δ*pbpG* | MG1655 *pbpG*:: *frt* | This study |
| MG1655Δ*ampH* | MG1655 *ampH*:: *frt* | This study |
| MG1655Δ*mrcA* | MG1655 *mrcA*:: *frt* | This study |
| MG1655Δ*mrcB* | MG1655 *mrcB*:: *frt* | This study |
| MG1655Δ*lpoA* | MG1655 *lpoA*:: *frt* | This study |
| MG1655Δ*lpoB* | MG1655 *lpoB*:: *frt* | This study |
| MG1655 chromosomal P_BAD_::*mepS* | MG1655 P_BAD_*::mepS-Flag Cm^r^* | This study |
| MG1655 chromosomal P_BAD_::*mepM* Δ*mepM* | MG1655 *mepM*::*frt* P_BAD_*::mepM-Flag Cm^r^* | This study |
| MG1655 chromosomal P_BAD_::*mepM* Δ*mepS* | MG1655 *mepS*::*frt* P_BAD_*::mepS-Flag Cm^r^* | This study |
| MG1655 chromosomal P_BAD_::*mepM* Δ*mepM* Δ*mrcA* | MG1655 *mepM*::*frt mrcA*::*Km^r^* P_BAD_*::mepM-Flag Cm^r^* | This study |
| MG1655 chromosomal P_BAD_::*mepM* Δ*mepM* Δ*mrcB* | MG1655 *mepM*::*frt mrcB*::*Km^r^* P_BAD_*::mepM-Flag Cm^r^* | This study |
| MG1655 chromosomal P_BAD_::*mepS* Δ*mepS* Δ*mrcA* | MG1655 *mepS*::*frt mrcA*::*Km^r^* P_BAD_*::mepS-Flag Cm^r^* | This study |
| MG1655 chromosomal P_BAD_::*mepS* Δ*mepS* Δ*mrcB* | MG1655 *mepS*::*frt mrcB*::*Km^r^* P_BAD_*::mepS-Flag Cm^r^* | This study |
| MG1655 chromosomal *mepM*-*3×Flag* | MG1655 *mepM*-*3×Flag Cm^r^* | This study |
| MG1655Δ*mepS* Δ*mrcA* | MG1655 *mepS*::*frt* *mrcA*::*Km^r^* | This study |
| MG1655Δ*mepS* Δ*mrcB* | MG1655 *mepS*::*frt mrcB*::*Km^r^* | This study |
| MG1655Δ*mepS* Δ*mepH* | MG1655 *mepS*::*frt mepH*::*Km^r^* | This study |
| MG1655Δ*mepS* Δ*mepH* Δ*pbpG* | MG1655 *mepS*::*frt mepH*::*frt pbpG*::*Km^r^* | This study |
| MG1655Δ*mepM* Δ*mepS* | MG1655 *mepM*::*frt mepS*::*Km^r^* | This study |
| ER2566 | F-λ-*fhuA2* [*lon*] *ompT* *lacZ*::T7 gene 1 gal *sulA11* Δ(*mcrC*-*mrr*)*114*::*IS10* R(*mcr*-*73*::*miniTn10*-TetS)2 R(*zgb-210*::*Tn10*)(TetS) *endA1* [*dcm*] | New England Biolabs |
| **Plasmids** |  |  |
| pBAD24 | Expression vector under control of arabinose-inducible promoter, Amp^r^ | Addgene |
| pBAD24(km) | pBAD24-based expression vector, Km^r^ | This study |
| pBAD24(cm) | pBAD24-based expression vector, Cm^r^ | This study |
| pBAD24(tet) | pBAD24-based expression vector, Tet^r^ | This study |
| pKD13 | Template plasmid for the amplification of the kanamycin-resistance gene bordered by FRT sites, Km^r^ | (Datsenko and Wanner, 2000) |
| pKD46 | λ Red recombinase expression plasmid Ts replicon, Amp^r^ | (Datsenko and Wanner, 2000) |
| pCP20 | FLP helper plasmid Ts replicon, Amp^r^, Cm^r^ | (Datsenko and Wanner, 2000) |
| pET28a | Expression vector under control of T7 promoter, Km^r^ | Novagen |
| pET24a | Expression vector under control of T7 promoter, Km^r^ | Novagen |
| pBAD-MepA | pBAD24(cm)-based expression vector for MepA, Cm^r^ | This study |
| pBAD-MepH | pBAD24(cm)-based expression vector for MepH, Cm^r^ | This study |
| pBAD(tet)-MepH | pBAD24(tet)-based expression vector for MepH, Tet^r^ | This study |
| pBAD-MepM | pBAD24(cm)-based expression vector for MepM, Cm^r^ | This study |
| pBAD-MepS | pBAD24(cm)-based expression vector for MepS, Cm^r^ | This study |
| pBAD(km)-MepS | pBAD24(km)-based expression vector for MepS, Km^r^ | This study |
| pBAD-DacB | pBAD24(cm)-based expression vector for DacB, Cm^r^ | This study |
| pBAD-PbpG | pBAD24(cm)-based expression vector for PbpG, Cm^r^ | This study |
| pBAD(tet)-PbpG | pBAD24(tet)-based expression vector for PbpG, Tet^r^ | This study |
| pBAD-AmpH | pBAD24(cm)-based expression vector for AmpH, Cm^r^ | This study |
| pBAD-PBP1a | pBAD24(cm)-based expression vector for PBP1a, Cm^r^ | This study |
| pBAD-PBP1b | pBAD24(cm)-based expression vector for PBP1b, Cm^r^ | This study |
| pBAD-LpoA | pBAD24(cm)-based expression vector for LpoA, Cm^r^ | This study |
| pBAD-LpoB | pBAD24(cm)-based expression vector for LpoB, Cm^r^ | This study |
| pBAD-MepM(ΔlytM) | pBAD24(cm)-based expression vector for MepM without LytM domain (313–440 amino acids), Cm^r^ | This study |
| pBAD-MepM(H314A) | pBAD24(cm)-based expression vector for MepM(H314A), Cm^r^ | This study |
| pBAD-MepS(C94A) | pBAD24(cm)-based expression vector for MepS(C94A), Cm^r^ | This study |
| pBAD-Flag | pBAD24(cm)-based expression vector for the 3xFlag tag, Cm^r^ | This study |
| pBAD-MepM-Flag | pBAD24(cm)-based expression vector for MepM fused with the 3xFlag tag at the C-terminus, Cm^r^ | This study |
| pBAD-MepM(ΔLytM)-Flag | pBAD24(cm)-based expression vector for MepM(ΔLytM) fused with the 3xFlag tag at the C-terminus, Cm^r^ | This study |
| pBAD-MepM(H314A)-Flag | pBAD24(cm)-based expression vector for MepM(H314A) fused with the 3xFlag tag at the C-terminus, Cm^r^ | This study |
| pBAD-MepS-Flag | pBAD24(cm)-based expression vector for MepS fused with the 3xFlag tag at the C-terminus, Cm^r^ | This study |
| pBAD-MepS(S28D&R29D)-Flag | pBAD24(cm)-based expression vector for MepS(S28D&R29D) fused with the 3xFlag tag at the C-terminus, Cm^r^ | This study |
| pBAD-MepM(N)-Flag | pBAD24(cm)-based expression vector for the N-terminal transmembrane domain (1–40 amino acids) of MepM and the 3xFlag tag, Cm^r^ | This study |
| pBAD-MepS(N)-Flag | pBAD24(cm)-based expression vector for the N-terminal region (1–27 amino acids) of MepS and the 3xFlag tag, Cm^r^ | This study |
| pBAD-DsbA(N)-Flag | pBAD24(cm)-based expression vector for the signal sequence (1–19 amino acids) of DsbA and the 3xFlag tag, Cm^r^ | This study |
| pBAD-DsbA(N)-MepM-Flag | pBAD24(cm)-based expression vector for MepM fused with the signal sequence of DsbA at the N-terminus and the 3xFlag tag at the C-terminus, Cm^r^ | This study |
| pBAD-MepS(N)-MepM-Flag | pBAD24(cm)-based expression vector for MepM fused with the N-terminal region of MepS at the N-terminus and the 3xFlag tag at the C-terminus, Cm^r^ | This study |
| pBAD-DsbA(N)-MepS-Flag | pBAD24(cm)-based expression vector for MepS fused with the signal sequence of DsbA at the N-terminus and the 3xFlag tag at the C-terminus, Cm^r^ | This study |
| pBAD-MepM(N)-MepA-Flag | pBAD24(cm)-based expression vector for MepA fused with the N-terminal transmembrane domain of MepM at the N-terminus and the 3xFlag tag at the C-terminus, Cm^r^ | This study |
| pBAD-MepM(N)-MepH-Flag | pBAD24(cm)-based expression vector for MepH fused with the N-terminal transmembrane domain of MepM at the N-terminus and the 3xFlag tag at the C-terminus, Cm^r^ | This study |
| pBAD-MepM(N)-MepS-Flag | pBAD24(cm)-based expression vector for MepS fused with the N-terminal transmembrane domain of MepM at the N-terminus and the 3xFlag tag at the C-terminus, Cm^r^ | This study |
| pBAD-MepM(N)-DacB-Flag | pBAD24(cm)-based expression vector for BacB fused with the N-terminal transmembrane domain of MepM at the N-terminus and the 3xFlag tag at the C-terminus, Cm^r^ | This study |
| pBAD-MepM(N)-PbpG-Flag | pBAD24(cm)-based expression vector for PbpG fused with the N-terminal transmembrane domain of MepM at the N-terminus and the 3xFlag tag at the C-terminus, Cm^r^ | This study |
| pBAD-MepM(N)-AmpH-Flag | pBAD24(cm)-based expression vector for AmpH fused with the N-terminal transmembrane domain of MepM at the N-terminus and the 3xFlag tag at the C-terminus, Cm^r^ | This study |
| pBAD-MepS(N)-MepA-Flag | pBAD24(cm)-based expression vector for MepA fused with the N-terminal region of MepS at the N-terminus and the 3xFlag tag at the C-terminus, Cm^r^ | This study |
| pBAD-MepS(N)-MepH-Flag | pBAD24(cm)-based expression vector for MepH fused with the N-terminal region of MepS at the N-terminus and the 3xFlag tag at the C-terminus, Cm^r^ | This study |
| pBAD-MepS(N)-DacB-Flag | pBAD24(cm)-based expression vector for DacB fused with the N-terminal region of MepS at the N-terminus and the 3xFlag tag at the C-terminus, Cm^r^ | This study |
| pBAD-MepS(N)-PbpG-Flag | pBAD24(cm)-based expression vector for PbpG fused with the N-terminal region of MepS at the N-terminus and the 3xFlag tag at the C-terminus, Cm^r^ | This study |
| pBAD-MepS(N)-AmpH-Flag | pBAD24(cm)-based expression vector for AmpH fused with the N-terminal region of MepS at the N-terminus and the 3xFlag tag at the C-terminus, Cm^r^ | This study |
| pBAD-MepM(N)-EnvC-Flag | pBAD24(cm)-based expression vector for EnvC fused with the N-terminal transmembrane domain of MepM at the N-terminus and the 3xFlag tag at the C-terminus, Cm^r^ | This study |
| pBAD-MepM(N)-NlpD-Flag | pBAD24(cm)-based expression vector for NlpD fused with the N-terminal transmembrane domain of MepM at the N-terminus and the 3xFlag tag at the C-terminus, Cm^r^ | This study |
| pBAD-MepM(N)-YgeR-Flag | pBAD24(cm)-based expression vector for YgeR fused with the N-terminal transmembrane domain of MepM at the N-terminus and the 3xFlag tag at the C-terminus, Cm^r^ | This study |
| pBAD-MepM(ΔC)-Flag | pBAD24(cm)-based expression vector for MepM(ΔC) fused with the 3xFlag tag at the C-terminus, Cm^r^ | This study |
| pBAD-MepM(LysM::LysM^NlpD^)-Flag | pBAD24(cm)-based expression vector for MepM(LysM::LysM^NlpD^) fused with the N-terminal transmembrane domain of MepM at the N-terminus and the 3xFlag tag at the C-terminus, Cm^r^ | This study |
| pBAD-MepM(LytM::NlpC/P60^MepS^)-Flag | pBAD24(cm)-based expression vector for MepM(LytM::NlpC/P60^MepS^) fused with the 3xFlag tag at the C-terminus, Cm^r^ | This study |
| pBAD-MepS(ΔC)-Flag | pBAD24(cm)-based expression vector for MepS(ΔC) fused with the 3xFlag tag at the C-terminus, Cm^r^ | This study |
| pBAD-MepS(NlpC/P60::NlpC/P60^MepH^)-Flag | pBAD24(cm)-based expression vector for MepS(NlpC/P60::NlpC/P60^MepH^) fused with the 3xFlag tag at the C-terminus, Cm^r^ | This study |
| pBAD-MepS(N)-MepH(NlpC/P60::NlpC/P60^MepS^)-Flag | pBAD24(cm)-based expression vector for MepH(NlpC/P60::NlpC/P60^MepS^) fused with the N-terminal region of MepS at the N-terminus and the 3xFlag tag at the C-terminus, Cm^r^ | This study |
| pET24a-MepM | pET24a-based expression vector for MepM with C-terminal 6 histidines, Km^r^ | This study |
| pET24a-MepM(H314A) | pET24a-based expression vector for MepM(H314A) with C-terminal 6 histidines, Km^r^ | This study |
| pET28a-MepS | pET28a-based expression vector for MepS with N-terminal 6 histidines, Km^r^ | This study |

**Supplementary Table S2.** **Oligonucleotides used in this study**

| **Name** | **Oligonucleotide sequence (5’–3’)** | **Use(s)** |
| --- | --- | --- |
| MepA-FRT-F | CCGCGATTGCGCTGCTGGCTCTGCTTGCCAGTAGCGCCAGCCTGGCAGCGGTGTAGGCTGGAGCTGCTTC | Deletion |
| MepA-FRT-R | TGTTCCCGGTTTTGGAGGTTCAAACCAGCTTTGCAGTTCTGCCCCGCAACATTCCGGGGATCCGTCGACC |  |
| MepH-FRT-F | TGTAAATCAGACGCAGGCATGATAGACCTGCCTTTACAGAGGGACGCTCAGTGTAGGCTGGAGCTGCTTC |  |
| MepH-FRT-R | GCCGACATACACGCCGACATGATCGGCTGTGCCGCGTCCCTGAGTACGGAATTCCGGGGATCCGTCGACC |  |
| MepM-FRT-F | CCAACCAGTATGCGAGCTGCCTGAAAGGAGATTAATGAGGAAGTGATTACGTGTAGGCTGGAGCTGCTTC |  |
| MepM-FRT-R | GACGAGATACGGAACTGTTTCGCCGTCGGGAATCGCAAGAATCCTTTCGCATTCCGGGGATCCGTCGACC |  |
| MepS-FRT-F | TTTTTTATAACGATATTTGTCGTTAAGGACTTCAAGGGAAAACAAACAACGTGTAGGCTGGAGCTGCTTC |  |
| MepS-FRT-R | CTTTCAGTTAACTCGTCAGGATAGCCAAGGGATTGCATCCAAACGGTTTAATTCCGGGGATCCGTCGACC |  |
| DacB-FRT-F | GCGTTGCGCCGTAGTATGACGGCTCGATTCCAGGTTGTTAGCGCGAGATTGTGTAGGCTGGAGCTGCTTC |  |
| DacB-FRT-R | TCGTGCAGCGGGGCCGACTGTTTACTGGCAACTACCGTTCCAGGTTCGTTATTCCGGGGATCCGTCGACC |  |
| PbpG-FRT-F | CTCCGGCGGTGCGCAACCCGTGCGCGTGAACCACTATCTGAATGCTCATCGTGTAGGCTGGAGCTGCTTC |  |
| PbpG-FRT-R | ATTTGCCAAACGCGTCCATCACTACCAGCGCCACCGGTTTATTATTGATAATTCCGGGGATCCGTCGACC |  |
| AmpH-FRT-F | AGTAGTATAAATACGCTCAGTTACCTTCATTCAATCTATGGACACCACCGGTGTAGGCTGGAGCTGCTTC |  |
| AmpH-FRT-R | ATCACTTTCGTAAACTGCGCGCGCTGATAAATCAACGTCTGCATGCGATCATTCCGGGGATCCGTCGACC |  |
| MrcA-FRT-F | CGGATGTGGCGACATTAAAAGATGTTCGCCTGCAAATTCCGATGCAGATTGTGTAGGCTGGAGCTGCTTC |  |
| MrcA-FRT-R | ACAAGTGCACTTTGTCAGCAAACTGAAAAGGCGCCGAAGCGCCTTTTTAAATTCCGGGGATCCGTCGACC |  |
| MrcB-FRT-F | AGAAGAACAGAAAATCGGGCTTTTGCGCCTGAATATTGCGGAGAAAAAGCGTGTAGGCTGGAGCTGCTTC |  |
| MrcB-FRT-R | TGTTATTTTACCGGATGGCAACTCGCCATCCGGTATTTCACGCTTAGATGATTCCGGGGATCCGTCGACC |  |
| LpoA-FRT-F | TGCAGCAAAGCTCTGATGATACCAGGATCAACTGGCAATTACTCGCCATTGTGTAGGCTGGAGCTGCTTC |  |
| LpoA-FRT-R | CGTTATTCACCGCGCTGAGTGCCTGCTGCATTAACGGTAGATTACCGCCTATTCCGGGGATCCGTCGACC |  |
| LpoB-FRT-F | TTTGTAAGGGGTGAATCTTGATGACAAAAATGAGTCGCTACGCCTTGATTGTGTAGGCTGGAGCTGCTTC |  |
| LpoB-FRT-R | ATTGCGGGCAATGCCTATGGCTTTACTACGGGTGCCTAAACTGTCCTGCGATTCCGGGGATCCGTCGACC |  |
| MepA-cfm-F | TTCCACGCTGGTAAAAAATGAATAAAACCG | Deletion confirm |
| MepA-cfm-R | CGGAGGCGGTGTCTTCTTCTCAGGCTTTGT |  |
| MepH-cfm-F | CTTCTTCTATGCATTAGAATCATCAAGTTT |  |
| MepH-cfm-R | TGTGCGCGGTGACTGAATAAATTTGCCGTT |  |
| MepM-cfm-F | CAAGCTATTCAGAATTCCTGAGTCAATTAG |  |
| MepM-cfm-R | ACCGGATTAGTACGACGCGGGTTAAAATTA |  |
| MepS-cfm-F | CCAGGTAATTAGTCTCGTGTCGCTTGGCAT |  |
| MepS-cfm-R | TGAAAACAAAAAAGCACTGCCTAAGCAGTG |  |
| DacB-cfm-F | TTCCCGAAGGGTCATCGTTTACTTTATAGG |  |
| DacB-cfm-R | TCCGACTTTTTCAGCATAATCTTAAGCAGA |  |
| PbpG-cfm-F | GATGGCGTCGCTAGCCTCAGTAAATCCTTA |  |
| PbpG-cfm-R | GCAGGCGGCTGGCATCGGCAAAATGGGTAT |  |
| AmpH-cfm-F | CACTAATTCGCCCTTTGCAATCTATCAATG |  |
| AmpH-cfm-R | GCATCCGCTTTGCCGGGGACATCCATGCCA |  |
| MrcA-cfm-F | GCCTATACCGCTACATCGAGCCACAACTGC |  |
| MrcA-cfm-R | TAACGCGTTCACGCCGTATCCGGCATAAAC |  |
| MrcB-cfm-F | TTTGAGAGATATCTTCTTCTGTCTTGTAAC |  |
| MrcB-cfm-R | GAAAAGAAAGGGTTAATATCTTAGATGGGA |  |
| LpoA-cfm-F | GTGTTGTTTCATTGTGTCGTCCGTATTGCC |  |
| LpoA-cfm-R | ATAAACCGCAGTCCTTTGCCTTCCAGCCAG |  |
| LpoB-cfm-F | GTAACGCTGTACGGCAGCGCCAATTTTCTG |  |
| LpoB-cfm-R | CGTGACGGGATGATACTGCGGGAAAAAGCG |  |
| Chromosomal pBAD insertion-F | ACAAGCTTAAAAAATATACTGTTATTCTAAAGAAAAAATTAACAGGCATTAAGAAACCAATTGTCCATAT | Chromosomal insertion |
| Chromosomal pBAD insertion-R | ACAATTATCGATACAAAAAATTAAATTTAATCAAAGTGTTATTTGTATGAATCACCCAAATCAAGTTTTT |  |
| Chromosomal pBAD insertion-cfm-F | AGAACTATTCATCAACAGCGTATAGAGGCG | Chromosomal insertion  cfm |
| Chromosomal pBAD insertion-cfm-R | GATAAACCTGTGTTTGCGGCATTAACGCTC |  |
| Chromosomal MepM-3*×*Flag-F | GCGCTTTCCG GTAATACCGG ACGTTCAACC GGGCCGCATC | Chromosomal MepM-3*×*Flag |
| Chromosomal MepM-3*×*Flag-R | GTGTAGCGGCGCAACTTGCCCCGCACCAAATAAAAAAAGCCGGTACTGACTGCGTACCGGCTGCGAATGGTTACGCCCCGCCCTGCCACT |  |
| Chromosomal MepM-3*×*Flag-cfm-F | CGTTATATGCACTTGCGCAAGATTCTGGTG | Chromosomal MepM-3*×*Flag  cfm |
| Chromosomal MepM-3*×*Flag-cfm-R | GTTTCCATGCTTTTCCAGTTTCGGATAAGG |  |
| pBAD24-MepA-F | CTAGCAGGAGGAATTCATGAATAAAACCGCGATTGC | pBAD24  cloning |
| pBAD24-MepA-R | GCAGGTCGACTCTAGATTCCATTAGATCACGTGCTC |  |
| pBAD24-MepH-F | CTAGCAGGAGGAATTCATGGCGCGGATAAACCG |  |
| pBAD24-MepH-R | GCAGGTCGACTCTAGAAGGGTAAAGTTTTAGCG |  |
| pBAD24-MepM-F | CTAGCAGGAGGAATTCATGCAACAGATAGCCCGCTC |  |
| pBAD24-MepM-R | GCAGGTCGACTCTAGATTAATCAAACCGTAGCTGCG |  |
| pBAD24-MepS-F | CTAGCAGGAGGAATTCATGGTCAAATCTCAACCG |  |
| pBAD24-MepS-R | GCAGGTCGACTCTAGAGTTTATTAGCTGCGGCTG |  |
| pBAD24-DacB-F | CTAGCAGGAGGAATTCATGCGATTTTCCAGA |  |
| pBAD24-DacB-R | GCAGGTCGACTCTAGATGACTAATTGTTCTG |  |
| pBAD24-PbpG-F | CTAGCAGGAGGAATTCATGCCGAAATTTCGAGTTTC |  |
| pBAD24-PbpG-R | GCAGGTCGACTCTAGACACTTAATCGTTCTGTGCCG |  |
| pBAD24-AmpH-F | CTAGCAGGAGGAATTCATGAAACGTAGTCTGC |  |
| pBAD24-AmpH-R | GCAGGTCGACTCTAGATAATCAGGACGCGGGG |  |
| pBAD24-PBP1a-F | CTAGCAGGAGGAATTCATGAAGTTCGTAAAGTATTT |  |
| pBAD24-PBP1a-R | GCAGGTCGACTCTAGATTTTTAATCAGAACAATTCC |  |
| pBAD24-PBP1b-F | CTAGCAGGAGGAATTCATGGCCGGGAATGACCGCGA |  |
| pBAD24-PBP1b-R | GCAGGTCGACTCTAGACGCCATCCGGTATTTCACGC |  |
| pBAD24-LpoA-F | CTAGCAGGAGGAATTCATGGTACCCTCAACATTTTC |  |
| pBAD24-LpoA-R | GCAGGTCGACTCTAGATTAACTGACGGGGACTACCT |  |
| pBAD24-LpoB-F | CTAGCAGGAGGAATTCATGACAAAAATGAGTCGCTA |  |
| pBAD24-LpoB-R | GCAGGTCGACTCTAGATTATTGCTGCGAAACGGCAC |  |
| MepS(N)-linker(S28D&R29D)-MepS-F | CTTTCTGCATGTGATGATAGTGCAAATAAC | Point mutants |
| MepS(N)-linker(S28D&R29D)-MepS-R | GTTATTTGCACTATCATCACATGCAGAAAG |  |
| MepM(H314A)-F | CGCGTTGCACCAGCCAGAGGTGTTGATTTCG |  |
| MepM(H314A)-R | CGAAATCAACACCTCTGGCTGGTGCAACGCG |  |
| MepS(C94A)-F | AAAAAGGTATCGATGCTTCTGGTTTCGTAC |  |
| MepS(C94A)-R | GTACGAAACCAGAAGCATCGATACCTTTTT |  |
| pBAD-Flag-F | CCTCTAGAGTCGACCTGCAGGATTATAAGGATGACGATGACAAAGCAGACTACAAAGACGACGATGATAAGGCTGATTATAAAGATGATGACGACAAAtagCTGCAGGCATGCAAGCTTGG | pBAD-Flag |
| pBAD-Flag-R | CCAAGCTTGCATGCCTGCAGctaTTTGTCGTCATCATCTTTATAATCAGCCTTATCATCGTCGTCTTTGTAGTCTGCTTTGTCATCGTCATCCTTATAATCCTGCAGGTCGACTCTAGAGG |  |
| pBAD24-MepM-F | CTAGCAGGAGGAATTCATGCAACAGATAGCCCGCTC | pBAD-MepM(ΔlytM) |
| MepM(ΔLytM)-R | GCAGGTCGACTCTAGATTATGCAACGCGACCGGTCA |  |
| pBAD24-MepM-F | CTAGCAGGAGGAATTCATGCAACAGATAGCCCGCTC | pBAD-MepM(N)-Flag |
| MepM(N)-_-FLAG-R | GCAGGTCGACTCTAGAAACATAAGGCCGCCAGACAG |  |
| pBAD24-MepS-F | CTAGCAGGAGGAATTCATGGTCAAATCTCAACCG | pBAD-MepS(N)-Flag |
| MepS(N)-_-FLAG-R | GCAGGTCGACTCTAGAACATGCAGAAAGCAGAACCG |  |
| pBAD24-DsbA-F | CTAGCAGGAGGAATTCATGAAAAAGATTTGGCTGGC | pBAD-DsbA(N)-Flag |
| DsbA(N)-_-FLAG-R | GCAGGTCGACTCTAGACGCCGATGCGCTAAACGCTA |  |
| DsbA(N)-MepM-F | CGCATCGGCGTCTAGATATCACCGTGATGCCACGCC | pBAD-DsbA(N)-MepM-Flag |
| MepM-FLAG-R | AATCCTGCAGGTCGACATCAAACCGTAGCTGCGGCA |  |
| MepS(N)-MepM-F | TTCTGCATGTTCTAGATATCACCGTGATGCCACGCC | pBAD-MepS(N)-MepM-Flag |
| MepM-FLAG-R | AATCCTGCAGGTCGACATCAAACCGTAGCTGCGGCA |  |
| DsbA(N)-MepS-F | CGCATCGGCGTCTAGAAGTGCAAATAACACCGCAAAG | pBAD-DsbA(N)-MepS-Flag |
| MepS-FLAG-R | AATCCTGCAGGTCGACGCTGCGGCTGAGAACCCGGC |  |
| MepM(N)-MepA-F | GCCTTATGTTTCTAGAGCGACGCCGTGGCAAAAAAT | pBAD-MepM(N)-MepA-Flag |
| MepA-FLAG-R | AATCCTGCAGGTCGACGATCACGTGCTCATCCAGTA |  |
| MepM(N)-MepH-F | GCCTTATGTTTCTAGATCAAAGCAAGCCAGGGAGAG | pBAD-MepM(N)-MepH-Flag |
| MepH-FLAG-R | AATCCTGCAGGTCGACGCGAAGTGTTTTTGGGGTCA |  |
| MepM(N)-MepS-F | GCCTTATGTTTCTAGAAGTGCAAATAACACCGCAAA | pBAD-MepM(N)-MepS-Flag |
| MepS-FLAG-R | AATCCTGCAGGTCGACGCTGCGGCTGAGAACCCGGC |  |
| MepM(N)-DacB-F | GCCTTATGTTTCTAGAGCAAATGTTGATGAGTACAT | pBAD-MepM(N)-DacB-Flag |
| DacB-FLAG-R | AATCCTGCAGGTCGACATTGTTCTGATAAATATCTT |  |
| MepM(N)-PbpG-F | GCCTTATGTTTCTAGAGCCGCTACCACCGCTTCACA | pBAD-MepM(N)-PbpG-Flag |
| PbpG-FLAG-R | AATCCTGCAGGTCGACATCGTTCTGTGCCGTCTGCC |  |
| MepM(N)-AmpH-F | GCCTTATGTTTCTAGAGCGCAGCCGATCACTGAACC | pBAD-MepM(N)-AmpH-Flag |
| AmpH-FLAG-R | AATCCTGCAGGTCGACGGACGCGGGGATAACCAACG |  |
| MepS(N)-MepA-F | TTCTGCATGTTCTAGAGCGACGCCGTGGCAAAAAATAAC | pBAD-MepS(N)-MepA-Flag |
| MepA-FLAG-R | AATCCTGCAGGTCGACGATCACGTGCTCATCCAGTA |  |
| MepS(N)-MepH-F | TTCTGCATGTTCTAGATCAAAGCAAGCCAGGGAGAG | pBAD-MepS(N)-MepH-Flag |
| MepH-FLAG-R | AATCCTGCAGGTCGACGCGAAGTGTTTTTGGGGTCA |  |
| MepS(N)-DacB-F | TTCTGCATGTTCTAGAGCAAATGTTGATGAGTACAT | pBAD-MepS(N)-DacB-Flag |
| DacB-FLAG-R | AATCCTGCAGGTCGACATTGTTCTGATAAATATCTT |  |
| MepS(N)-PbpG-F | TTCTGCATGTTCTAGAGCCGCTACCACCGCTTCACAAC | pBAD-MepS(N)-PbpG-Flag |
| PbpG-FLAG-R | AATCCTGCAGGTCGACATCGTTCTGTGCCGTCTGCC |  |
| MepS(N)-AmpH-F | TTCTGCATGTTCTAGAGCGCAGCCGATCACTGAACCGG | pBAD-MepS(N)-AmpH-Flag |
| AmpH-FLAG-R | AATCCTGCAGGTCGACGGACGCGGGGATAACCAACG |  |
| MepM(N)-EnvC-F | GCCTTATGTTTCTAGAGATGAGCGTGACCAACTCAA | pBAD-MepM(N)-EnvC-Flag |
| EnvC-FLAG-R | AATCCTGCAGGTCGACTCTTCCCAACCACGGCTGTG |  |
| MepM(N)-NlpD-F | GCCTTATGTTTCTAGATGTTCTGACACTTCAAATCC | pBAD-MepM(N)-NlpD-Flag |
| NlpD-FLAG-R | AATCCTGCAGGTCGACTCGCTGCGGCAAATAACGCA |  |
| MepM(N)-YgeR-F | GCCTTATGTTTCTAGATCGGGTAGCAAATCATCCGA | pBAD-MepM(N)-YgeR-Flag |
| YgeR-FLAG-R | AATCCTGCAGGTCGACGCATTTTGGCTTGCTGCCCT |  |
| MepM(N)-MepM-F | GCCTTATGTTTCTAGATATCACCGTGATGCCACGCC | pBAD-MepM(ΔC)-Flag |
| MepM(ΔC)-FLAG-R | AATCCTGCAGGTCGACCGGGTTTACGGCCTGCTGGT |  |
| MepM(N)-NlpD-F | GCCTTATGTTTCTAGATGTTCTGACACTTCAAATCC | pBAD-MepM(LysM::LysM^NlpD^)-Flag |
| NlpD(ΔLytM)-MepM(LytM)-R | ACCTCTGTGTGGGCCTCCCTCAGAAGCGCC |  |
| NlpD(ΔLytM)-MepM(LytM)-F | AGGGAGGCCCACACAGAGGTGTTGATTTCG |  |
| MepM-FLAG-R | AATCCTGCAGGTCGACATCAAACCGTAGCTGCGGCA |  |
| MepM(N)-MepM-F | GCCTTATGTTTCTAGATATCACCGTGATGCCACGCC | pBAD-MepM(LytM::NlpC/P60^MepS^)-Flag |
| MepM(ΔLytM)-MepS(NlpC/P60)-R | AACGTACGCCTGCAACGCGACCGGTCACCG |  |
| MepM(ΔLytM)-MepS(NlpC/P60)-F | TCGCGTTGCAGGCGTACGTTATCGTCTGGG |  |
| MepS(NlpC/P60)-MepM(C)-R | CGCGGCAGTTTTGCCGTCAGCCGGCGTGCTTCGTTGTAAC |  |
| MepM(C)FLAG-F | CTGACGGCAAAACTGCCGCGTACCGAAGGG |  |
| MepM-FLAG-R | AATCCTGCAGGTCGACATCAAACCGTAGCTGCGGCA |  |
| MepS(N)-MepS-F | TTCTGCATGTTCTAGAAGTGCAAATAACACCGCAAAG | pBAD-MepS(ΔC)-Flag |
| MepS(ΔC)-FLAG-R | AATCCTGCAGGTCGACCCGGCGTGCTTCGTTGTAAC |  |
| MepH(NlpC/P60)-MepS(C)-F | CGCTCGTCGGGTTCTCAGCCGCAGCGTCGA | pBAD-MepS(NlpC/P60::NlpC/P60^MepH^)-Flag |
| MepS(ΔNlpC/P60)-MepH(NlpC/P60)-R | ATGGCTTACCTTTCCAGTCAGCATACTGAT |  |
| MepS(ΔNlpC/P60)-MepH(NlpC/P60)-F | TGACTGGAAAGGTAAGCCATATCGTTGGGG |  |
| MepH(NlpC/P60)-MepS(C)-R | GGCTGAGAACCCGACGAGCGCCAACATAGT |  |
| MepS(N)-MepH-F | TTCTGCATGTTCTAGATCAAAGCAAGCCAGGGAGAG | pBAD-MepS(N)-MepH(NlpC/P60::NlpC/P60^MepS^)-Flag |
| MepH(ΔNlpC/P60)-MepS(NlpC/P60)-R | AACGTACGCCAATTTGCTGCATCAGTTTAT |  |
| MepH(ΔNlpC/P60)-MepS(NlpC/P60)-F | GCAGCAAATTGGCGTACGTTATCGTCTGGG |  |
| MepS-FLAG-R | AATCCTGCAGGTCGACGCTGCGGCTGAGAACCCGGC |  |
| pET24-MepM(ΔTM)-F | AAGGAGATATACATATGTATCACCGTGATGCCACGCC | pET24a-MepM |
| pET24-MepM-R | CAAGCTTGTCGACGGAGCTCGAATCAAACCGTAGCTGCGGCA |  |
| pET28-MepS(ΔSS)-F | CGCGCGGCAGCCATATGAGTGCAAATAACACCGCAAA | pET28a-MepS |
| pET28-MepS-R | GCTCGAATTCGGATCCTCCAAACGGTTTATTAGCTG |  |

**References**

Blattner, F. R., Plunkett, G., 3rd, Bloch, C. A.*, et al.* (1997). The complete genome sequence of *Escherichia coli* K-12. *Science*. 277, 1453-1462. doi:

Datsenko, K. A., Wanner, B. L. (2000). One-step inactivation of chromosomal genes in *Escherichia coli* K-12 using PCR products. *Proc. Natl. Acad. Sci. USA*. 97, 6640-6645. doi: 10.1073/pnas.120163297
